# Development of dual‐inducible duet‐expression vectors for tunable gene expression control and CRISPR interference‐based gene repression in *Pseudomonas putida* KT2440

**DOI:** 10.1111/1751-7915.13832

**Published:** 2021-05-19

**Authors:** Rahul Gauttam, Aindrila Mukhopadhyay, Blake A. Simmons, Steven W. Singer

**Affiliations:** ^1^ The Joint BioEnergy Institute Emeryville CA USA; ^2^ Biological Systems and Engineering Division Lawrence Berkeley National Laboratory Berkeley CA USA

## Abstract

The development of *P. putida* as an industrial host requires a sophisticated molecular toolbox for strain improvement, including vectors for gene expression and repression. To augment existing expression plasmids for metabolic engineering, we developed a series of dual‐inducible duet‐expression vectors for *P. putida* KT2440. A number of inducible promoters (P*_lac_*, P*_tac_*, P*_tetR/tetA_* and P*_bad_*) were used in different combinations to differentially regulate the expression of individual genes. Protein expression was evaluated by measuring the fluorescence of reporter proteins (GFP and RFP). Our experiments demonstrated the use of compatible plasmids, a useful approach to coexpress multiple genes in *P. putida* KT2440. These duet vectors were modified to generate a fully inducible CRISPR interference system using two catalytically inactive Cas9 variants from *S. pasteurianus* (dCas9) and *S. pyogenes* (spdCas9). The utility of developed CRISPRi system(s) was demonstrated by repressing the expression of nine conditionally essential genes, resulting in growth impairment and prolonged lag phase for *P. putida* KT2440 growth on glucose. Furthermore, the system was shown to be tightly regulated, tunable and to provide a simple way to identify essential genes with an observable phenotype.

## Graphical abstract

To augment existing expression plasmids for metabolic engineering, we developed a series of dual‐inducible duet‐expression vectors for *P. putida* KT2440. These duet vectors were modified to generate a fully inducible CRISPR interference system using two catalytically inactive Cas9 variants from *S. pasteurianus* (dCas9) and *S. pyogenes* (spdCas9). The utility of developed CRISPRi system(s) was demonstrated by repressing the expression of nine conditionally essential genes, resulting in growth impairment and prolonged lag phase for *P. putida* KT2440 growth on glucose.

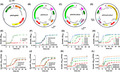

## Introduction

The last few years have seen many advances in systems metabolic engineering strategies to develop microbial hosts as cell factories for biotechnological applications. For decades, *Escherichia coli* has been the preferred microbial host for bioproduction and a robust platform for developing advanced genome engineering tools to aid in fundamental and applied research (Rosano *et al*., 2019). However, *E. coli* has some limitations and cannot be used as an optimal production host for every biotechnological application in the industry (de Lorenzo and Schmidt, 2018). Some microorganisms are known to possess innate mechanisms to tolerate harsh environmental stresses and may be developed as alternate model organisms (Calero and Nikel, 2019).

*Pseudomonas putida* KT2440 is a non‐pathogenic, Gram‐negative, obligately aerobic, soil bacterium that has been widely considered a potential industrial host for bioproduct formation owing to the diverse metabolic pathways that provide the bacterium with distinguished characteristics such as fast growth, high productivity, broad carbon source utilization, ability to cope up with redox stress and resistance to organic solvents (Nikel and de Lorenzo, 2018; Volke *et al*., 2020). These characteristics establish *P. putida* as a candidate for bioremediation applications and lignocellulosic biomass conversion into valuable products compared to model organisms (*E. coli*, *Bacillus* spp.) that cannot tolerate or consume toxic compounds generated during lignocellulosic pretreatment. Interestingly, the glucose metabolism does not proceed via the traditional Embden–Meyerhof–Parnas (EMP) pathway due to the absence of glycolytic enzyme phosphofructokinase (Pfk), and the bacterium harbours an alternate glycolytic pathway, the EDEMP cycle, to perform this function (Nikel and de Lorenzo, 2018). The ED in EDEMP stands for Entner–Doudoroff, and the majority of glucose catabolism proceeds through the ED route. The EDEMP cycle favours NADPH formation as opposed to ATP generation in the traditional glycolytic route, and NADPH plays a pivotal role during biocatalytic processes by providing a reducing environment and also counteracting environmental stress (Yu *et al*., 2018).

Advanced tools to introduce genomic manipulations focusing on systems metabolic engineering are rapidly being developed for various hosts. These developments combine synthetic biology and evolutionary engineering tools with classical metabolic engineering (Choi *et al*., 2019). Plasmid vectors are critical in both fundamental and applied studies (Nora *et al*., 2019). For *P. putida*, many expression systems have been developed to achieve its full potential, and new tools are continuously emerging for *Pseudomonas* engineering (Cook *et al*., 2018; Martinez‐Garcia and de Lorenzo, 2019). Two medium copy number origin of replications pBBR1 (30 ± 7) and pRO1600 (35 ± 5) are the most used for developing expression plasmids in *P. putida* KT2440. pBBR1 is a broad‐host‐range plasmid that consists of an *oriV* and a replication protein capable of replicating in a number of Gram‐negative bacteria, including *E. coli* and *P. putida* KT2440. pRO1600‐based plasmids harbour a hybrid of two origins (pRO1600/ColE1): the narrow‐host‐range ColE1 for replication in *E. coli* and pRO1600 (a plasmid from *P. aeruginosa* isolate) for replication in *P. putida* and closely related species (Silva‐Rocha *et al*., 2013; Volke *et al*., 2020). A user‐friendly Standard European Vector Architecture (SEVA) platform was created that facilitates swapping of genetic modules (e.g. promoters, replication origins and selection markers) to expand the options for large‐scale genome engineering in Gram‐negative bacteria (including *P. putida*) (Silva‐Rocha *et al*., 2013; Martinez‐Garcia et al., 2015). More recently, advanced CRISPR/(d)Cas9 technology has been adapted to use in *P. putida* to bring more robustness and control over gene expression (Aparicio *et al*., 2018; Sun *et al*., 2018; Tan *et al*., 2018; Banerjee *et al*., 2020; Batianis *et al*., 2020; Kim *et al*., 2020; Wirth *et al*., 2020). Two types of CRISPR‐Cas systems have been shown to repress genes in *P. putida* KT2440. Tan *et al*. (2018) explored the type II dCas9 homolog of *Streptococcus pasteurianus* (dCas9) for gene downregulation, which recognizes multiple protospacer adjacent motif (PAM) sequences, including 5'‐NNGTGA‐3' and 5'‐NNGCGA‐3'. In other studies, the use of a well‐characterized type II CRISPR‐Cas system from *Streptococcus pyogenes* (SpdCas9) that utilizes a shorter PAM site (5'‐NGG‐3') was explored for targeted gene repression in *P. putida* KT2440 (Sun *et al*., 2018; Batianis *et al*., 2020; Kim *et al*., 2020). Both CRISPRi approaches were shown to repress gene expression but were often accompanied by the leaky expression of dCas9 (or sgRNA) and resulted in gene downregulation even in the absence of an inducer. The success of a molecular toolbox is usually assessed by its ability to bring precise control over gene expression. When heterologously expressed proteins are toxic, it becomes essential to tightly control targeted gene expression by using inducible promoter systems (Martinez‐Garcia and de Lorenzo, 2017). Therefore, to modulate the gene expression, several inducible promoters have already been demonstrated in *P. putida*, including the native promoters, namely AlkS/P*_alkB_* (induction by di‐cyclopropyl ketone), XylS/P*_m_* (induction by methyl benzoate) and NahR/P*_sal_* (induction by salicylate) (Calero *et al*., 2016; Cook *et al*., 2018). The routinely used inducible promoter systems in *E. coli* such as *lacI*/P*_lac_* (IPTG‐inducible) and *tetR*/P*_tet_* (or *tetR*/P*_tetR/tetA_* both anhydrotetracycline inducible) were successfully adapted to use in *P. putida* (de Lorenzo *et al*., 1993; Chai *et al*., 2012). However, the efficacy of P*_tet_* in *Pseudomonas* species was questioned based on two contradicting reports, with one claiming to increase the expression levels by 38‐fold (Lee *et al*., 2011) while the other stated lower expression levels in the presence of inducer (Chai *et al*., 2012). Other inducible promoter systems have been developed in *P. putida*, including arabinose‐inducible *araC*/P*_bad_* (Calero *et al*., 2016), rhamnose‐inducible RhaRS/P*_rhaB_* (Calero *et al*., 2016), mannitol‐inducible MtlR/P*_mtlE_* (Hoffmann and Altenbuchner, 2015), hydroxypropionic acid‐inducible HpdR/P*_hpdH_* (Hanko *et al*., 2017), methyl ethyl ketone‐inducible MekR/P*_mekA_* (Graf and Altenbuchner, 2013) and cumate inducible CymR/P*_cym_* (Eaton, 1997), therefore providing an alternative to the routinely employed *lacI*/P*_lac_* and *tetR*/P*_tet_*. Most of these systems have some disadvantages that limit their use for gene expression studies: high basal expression (e.g. *lacI/*P*_lac_* and MtlR/P*_mtlE_*), low expression levels (e.g. *tetR*/P*_tet_*), use of toxic inducer (e.g. methyl ethyl ketone) and use of expensive substrate (e.g. L‐rhamnose) (Hoffmann and Altenbuchner, 2015; Martinez‐Garcia and de Lorenzo, 2017). In *P. putida*, the arabinose‐inducible promoter system (*araC*/P*_bad_*) satisfies most of the criteria that an ideal inducible system should have, such as high induction levels, tight regulation to prevent leakiness and specificity to an exogenous inducer to avoid cross‐talk among promoters (Bi *et al*., 2013). A major advantage of using arabinose‐inducible promoter (*araC*/P*_bad_*) is that *P. putida* KT2440 does not metabolize the inducer arabinose. To overcome the limitations presented by one inducible promoter system, researchers normally use two inducible systems simultaneously, in the form of either one plasmid (dual‐inducible approach) or two plasmids, each plasmid harbouring a different inducible promoter system (Gauttam *et al*., 2019a). Such an approach is useful in situations where one gene product is required to stimulate the expression of another gene, as in the case of co‐chaperone expression (Mueller *et al*., 2018). For this purpose, duet‐expression vectors were constructed in *Pseudomonas fluorescens* (Nakata, 2017), *P. putida* (Yu *et al*., 2018) and *Corynebacterium glutamicum* (Gauttam *et al*., 2019b). A dual‐inducible duet‐expression vector (pRGPDuo2) in *P. putida* was previously described, which separately regulates the expression of each gene by adjusting the amount of respective inducer (Gauttam *et al*., 2020).

In this study, a series of dual‐inducible duet‐expression shuttle vectors were constructed to control plasmid gene expression in *P. putida*. The functionality of these expression plasmids and compatibility for expressing recombinant proteins were characterized by measuring the expression of GFP and RFP in *P. putida* KT2440. Furthermore, we modified the duet vectors to develop fully inducible CRISPRi systems and demonstrated their activity by downregulating the expression of nine conditionally essential genes for *P*. *putida* KT2440 glucose growth.

## Results

### Functional validation of pBBR1‐derived duet‐expression vector pRGPDuo1 in *P. putida* KT2440

**Fig. 1:**
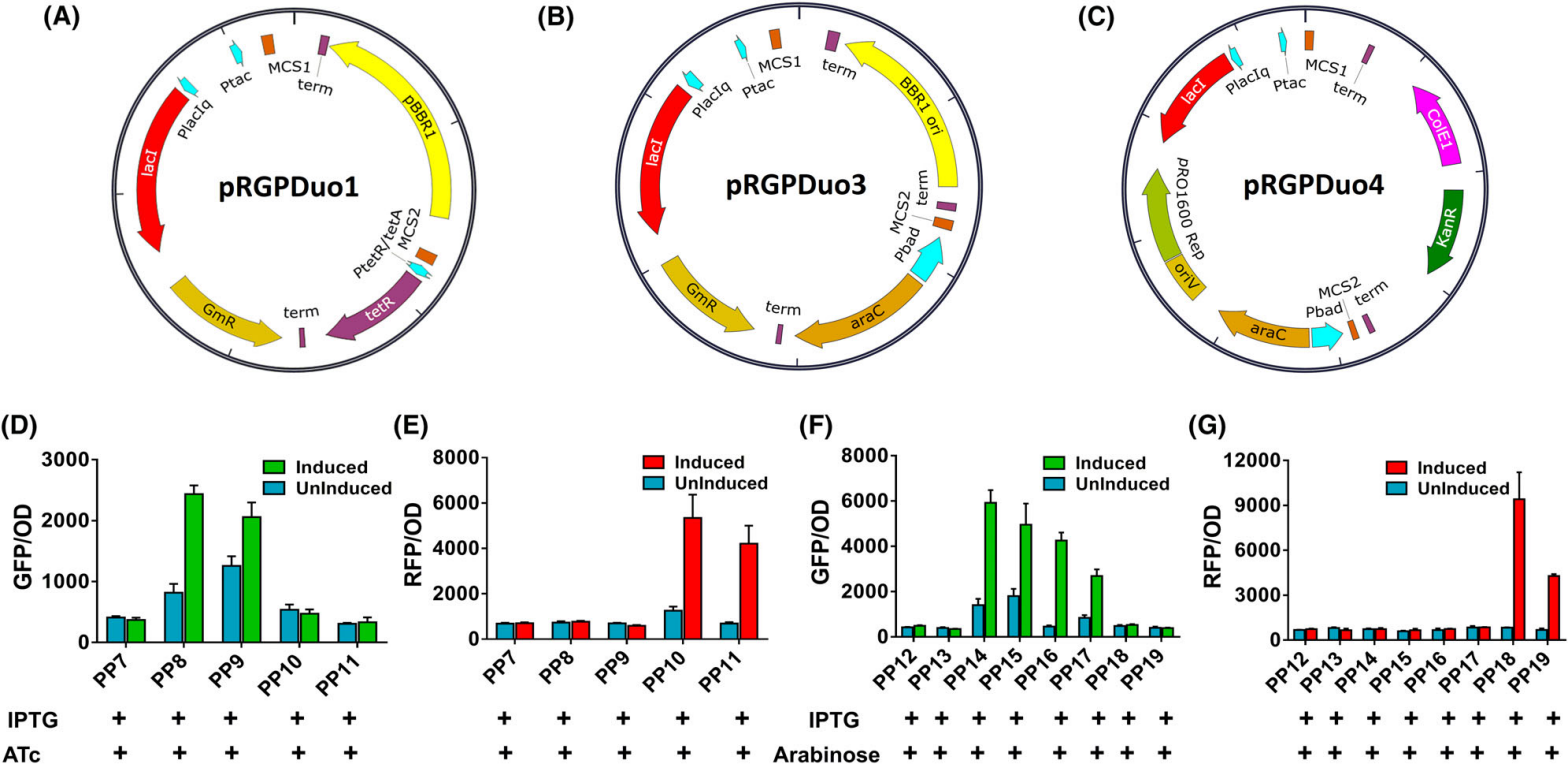
Vector maps of dual‐inducible duet‐expression vectors pRGPDuo1 (A), pRGPDuo3 (B) and pRGPDuo4 (C). The recombinant *P. putida* KT2440 strains were investigated for GFP (D, F) and RFP (E, G) fluorescence: PP7 (pRGPDuo1), PP8 (pRGPDuo1‐sfGFP_tet_), PP9 (pRGPDuo1‐sfGFP_tac_), PP10 (pRGPDuo1‐RFP_tet_), PP11 (pRGPDuo1‐RFP_tac_), PP12 (pRGPDuo3), PP13 (pRGPDuo4), PP14 (pRGPDuo3‐sfGFP_bad_), PP15 (pRGPDuo3‐sfGFP_tac_), PP16 (pRGPDuo4‐sfGFP_bad_), PP17 (pRGPDuo4‐sfGFP_tac_), PP18 (pRGPDuo4‐RFP_bad_) and PP19 (pRGPDuo4‐RFP_tac_). The graphs consist of relative fluorescence units normalized to OD_600_ during the mid‐exponential phase for each construct. Levels of fluorescence for uninduced (no inducer was added, blue bar in graphs) and induced cultures (with plasmid specific inducer combination: 1 mM IPTG, 1 μg ml^−1^ ATc and 0.2% w/v arabinose) are shown. The (+) sign corresponding to each inducer (below respective strain name) is indicative of the inducer added (to induced ones) to express the reporter gene depending on the promoter system (IPTG for P*_tac_*; ATc for P*_tetR/tetA_* and arabinose for P*_araC/bad_*). Data represent mean values of triplicate assays from at least two individual cultivations, and error bars represent standard deviations.

To investigate the functionality of expression vector pRGPDuo1 (Fig. 1A), the fluorescent genes (sfGFP and RFP) were introduced into MCS1 and MCS2 in different combinations to create vectors pRGPDuo1‐sfGFP_tet_, pRGPDuo1‐sfGFP_tac_, pRGPDuo1‐RFP_tet_ and pRGPDuo1‐RFP_tac_. For initial evaluation regarding plasmid's functionality, expression of one reporter gene was analysed per plasmid. The constructed plasmids were transformed into *P. putida* KT2440 strain to generate PP7‐11 (Table 1). The recombinant strains were grown in the presence of both inducers (1mM IPTG and 1 μg ml^−1^ ATc). Strain PP7 was used as control, and the respective uninduced counterpart(s) of the test strain(s) was used to determine the background fluorescence for GFP and RFP. As shown in Fig. 1D, GFP expression increased in PP8 and PP9 by 1.7‐ to threefold compared to their uninduced counterparts and by sixfold to eightfold compared to control strain PP7 when the cultures were induced. The higher background fluorescence of strains PP8 and PP9 in absence of inducers compared to control strain PP7 indicates leaky GFP expression from P*_tetR/tetA_* and P*_tac_* (Fig. 1D and Table S4). However, GFP expression was tightly controlled in PP8 (ATc‐inducible P_tetR/tetA_) compared to PP9 (IPTG‐inducible P*_tac_*). As expected, the fluorescence values in PP10 and PP11 were comparable to the values obtained for control strain PP7 (Fig. 1D). Similarly, RFP fluorescence was increased by fivefold to sixfold in recombinant strains PP10 and PP11 compared to their uninduced counterparts and sixfold to sevenfold compared to control strain PP7 when cultures were induced (Fig. 1E). The fluorescence levels in the uninduced PP11 were comparable to the control strain PP7. In contrast, the fluorescence levels in uninduced PP10 were increased by twofold, indicating leakiness of the repressor system (P*_tetR/tetA_*) (Fig. 1E and Table S4). RFP fluorescence was in the same range in strains PP8 and PP9 as uninduced counterparts for strains PP11 (Fig. 1E). Taken together, these data support the increase in expression levels for reporter gene in the test strains (compared to respective uninduced counterpart) cloned downstream of *lacI* controlled MCS1 as well as *tetR* controlled MCS2, which in turn establishes the functionality of vector pRGPDuo1 for gene expression studies in *P. putida* KT2440.

**Table 1:** Strains used in this study.

Strain^a^	Relevant characteristics	Source/reference
*Strains*		
*E. coli* DH5α	F^‐^ *φ*80*lacZ*∆M15 ∆(*lacZYA‐argF*) U169 *endA1 recA1 hsdR17* (r_k_^−^, m_k_^+^) *supE44 thi^‐1^ gyrA996 relA1 phoA*	Hanahan (1983)
*P. putida* KT2440	Wild type	ATCC 12633
*P. putida* PP7 (JPUB_014775)	*P. putida* carrying pRGPDuo1; Gent^R^	This study
*P. putida* PP8 (JPUB_014777)	*P. putida* carrying pRGPDuo1‐sfGFP_tet_; Gent^R^	This study
*P. putida* PP9 (JPUB_014779)	*P. putida* carrying pRGPDuo1‐sfGFP_tac_; Gent^R^	This study
*P. putida* PP10 (JPUB_014781)	*P. putida* carrying pRGPDuo1‐RFP_tet_; Gent^R^	This study
*P. putida* PP11 (JPUB_014783)	*P. putida* carrying pRGPDuo1‐RFP_tac_; Gent^R^	This study
*P. putida* PP12 (JPUB_014785)	*P. putida* carrying pRGPDuo3; Gent^R^	This study
*P. putida* PP13 (JPUB_014787)	*P. putida* carrying pRGPDuo4; Kan^R^	This study
*P. putida* PP14 (JPUB_014810)	*P. putida* carrying pRGPDuo3‐sfGFP_bad_; Gent^R^	This study
*P. putida* PP15 (JPUB_014789)	*P. putida* carrying pRGPDuo3‐sfGFP_tac_; Gent^R^	This study
*P. putida* PP16 (JPUB_014791)	*P. putida* carrying pRGPDuo4‐sfGFP_bad_; Kan^R^	This study
*P. putida* PP17 (JPUB_014793)	*P. putida* carrying pRGPDuo4‐sfGFP_tac_; Kan^R^	This study
*P. putida* PP18 (JPUB_014795)	*P. putida* carrying pRGPDuo4‐RFP_bad_; Kan^R^	This study
*P. putida* PP19 (JPUB_014797)	*P. putida* carrying pRGPDuo4‐RFP_tac_; Kan^R^	This study
*P. putida* PP20 (JPUB_014799)	*P. putida* carrying pRGPDuo1 + pRGPDuo2; Kan^R^ + Gent^R^	This study
*P. putida* PP21 (JPUB_014800)	*P. putida* carrying pRGPDuo3 + pRGPDuo4; Kan^R^ + Gent^R^	This study
*P. putida* PP22 (JPUB_014801)	*P. putida* carrying pRGPDuo1 + pRGPDuo4; Kan^R^ + Gent^R^	This study
*P. putida* PP23 (JPUB_014802)	*P. putida* carrying pRGPDuo2 + pRGPDuo3; Kan^R^ + Gent^R^	This study
*P. putida* PP24 (JPUB_014803)	*P. putida* carrying pRGPDuo1‐sfGFP_tac_ + pRGPDuo2‐RFP_tet_; Kan^R^ + Gent^R^	This study
*P. putida* PP25 (JPUB_014804)	*P. putida* carrying pRGPDuo2‐sfGFP_tac_ + pRGPDuo3‐RFP_bad_; Kan^R^ + Gent^R^	This study
*P. putida* PP26 (JPUB_014806)	*P. putida* carrying pRGPDuo2‐RFP_tet_ + pRGPDuo3‐sfGFP_bad_; Kan^R^ + Gent^R^	This study
*P. putida* PP27 (JPUB_014808)	*P. putida* carrying pRGPDuo2‐RFP_tet_ + pRGPDuo3‐sfGFP_tac_; Kan^R^ + Gent^R^	This study
*P. putida* PP28 (JPUB_014809)	*P. putida* carrying pRGPDuo3‐sfGFP_tac_ + pRGPDuo4‐RFP_bad_; Kan^R^ + Gent^R^	This study
*P. putida* PP31 (JPUB_018309)	*P. putida* KT2440 with deletion of *aceEF*	This study
*P. putida* PP34 (JPUB_018310)	*P. putida* KT2400 with genomic dCas9 integration	This study
*P. putida* PP35 (JPUB_018305)	*P. putida* PP34 carrying pRGPsgRNA; Kan^R^	This study
*P. putida* PP36 (JPUB_018307)	*P. putida* PP34 carrying pRGPsgRNA‐*aceE*; Kan^R^	This study
*P. putida* PP37 (JPUB_018311)	*P. putida* carrying pRGPdCas9; Kan^R^	This study
*P. putida* PP38 (JPUB_018315)	*P. putida* carrying pRGPdCas9‐*aceE*; Kan^R^	This study
*P. putida* PP39 (JPUB_018313)	*P. putida* carrying pRGPdCas9bad; Kan^R^	This study
*P. putida* PP40 (JPUB_018317)	*P. putida* carrying pRGPdCas9bad‐*aceE*; Kan^R^	This study
*P. putida* PP41 (JPUB_018319)	*P. putida* carrying pRGPdCas9bad‐*argB*; Kan^R^	This study
*P. putida* PP42 (JPUB_018321)	*P. putida* carrying pRGPdCas9bad‐*argH*; Kan^R^	This study
*P. putida* PP43 (JPUB_018323)	*P. putida* carrying pRGPdCas9bad‐*eda*; Kan^R^	This study
*P. putida* PP44 (JPUB_018325)	*P. putida* carrying pRGPdCas9bad‐*edd*; Kan^R^	This study
*P. putida* PP45 (JPUB_018327)	*P. putida* carrying pRGPdCas9bad‐*ftsZ*; Kan^R^	This study
*P. putida* PP46 (JPUB_018329)	*P. putida* carrying pRGPdCas9bad‐*pheA*; Kan^R^	This study
*P. putida* PP47 (JPUB_018331)	*P. putida* carrying pRGPdCas9bad‐*pyrF*; Kan^R^	This study
*P. putida* PP48 (JPUB_018333)	*P. putida* carrying pRGPdCas9bad‐*trpG*; Kan^R^	This study
*P. putida* PP49 (JPUB_018611)	*P. putida* carrying pRGPspdCas9bad; Kan^R^	This study
*P. putida* PP50 (JPUB_018612)	*P. putida* carrying pRGPspdCas9bad‐*aceE1*; Kan^R^	This study
*P. putida* PP51 (JPUB_018613)	*P. putida* carrying pRGPspCas9bad‐*aceE2*; Kan^R^	This study
*P. putida* PP52 (JPUB_018614)	*P. putida* carrying pRGPspdCas9bad‐*aceE3*; Kan^R^	This study
*P. putida* PP53 (JPUB_018587)	*P. putida* carrying pRGPspdCas9bad‐*aceE4*; Kan^R^	This study
*P. putida* PP54 (JPUB_018588)	*P. putida* carrying pRGPspdCas9bad‐*argB1*; Kan^R^	This study
*P. putida* PP55 (JPUB_018589)	*P. putida* carrying pRGPspdCas9bad‐*argH1*; Kan^R^	This study
*P. putida* PP56 (JPUB_018590)	*P. putida* carrying pRGPspdCas9bad‐*argH2*; Kan^R^	This study
*P. putida* PP57 (JPUB_018591)	*P. putida* carrying pRGPspdCas9bad‐*argH3*; Kan^R^	This study
*P. putida* PP58 (JPUB_018592)	*P. putida* carrying pRGPspdCas9bad‐*argH4*; Kan^R^	This study
*P. putida* PP59 (JPUB_018593)	*P. putida* carrying pRGPspdCas9bad‐*eda1*; Kan^R^	This study
*P. putida* PP60 (JPUB_018594)	*P. putida* carrying pRGPspdCas9bad‐*edd1*; Kan^R^	This study
*P. putida* PP61 (JPUB_018595)	*P. putida* carrying pRGPspdCas9bad‐*edd2*; Kan^R^	This study
*P. putida* PP62 (JPUB_018596)	*P. putida* carrying pRGPspdCas9bad‐*edd3*; Kan^R^	This study
*P. putida* PP63 (JPUB_018597)	*P. putida* carrying pRGPspdCas9bad‐*edd4*; Kan^R^	This study
*P. putida* PP64 (JPUB_018598)	*P. putida* carrying pRGPspdCas9bad‐*ftsZ1*; Kan^R^	This study
*P. putida* PP65 (JPUB_018599)	*P. putida* carrying pRGPspdCas9bad‐*pheA1*; Kan^R^	This study
*P. putida* PP66 (JPUB_018600)	*P. putida* carrying pRGPspdCas9bad‐*pheA2*; Kan^R^	This study
*P. putida* PP67 (JPUB_018601)	*P. putida* carrying pRGPspdCas9bad‐*pheA3*; Kan^R^	This study
*P. putida* PP68 (JPUB_018602)	*P. putida* carrying pRGPspdCas9bad‐*pheA4*; Kan^R^	This study
*P. putida* PP69 (JPUB_018603)	*P. putida* carrying pRGPspdCas9bad‐*pyrF1*; Kan^R^	This study
*P. putida* PP70 (JPUB_018604)	*P. putida* carrying pRGPspdCas9bad‐*pyrF2*; Kan^R^	This study
*P. putida* PP71 (JPUB_018605)	*P. putida* carrying pRGPspdCas9bad‐*pyrF3*; Kan^R^	This study
*P. putida* PP72 (JPUB_018606)	*P. putida* carrying pRGPspdCas9bad‐*pyrF4*; Kan^R^	This study
*P. putida* PP73 (JPUB_018607)	*P. putida* carrying pRGPspdCas9bad‐*trpG1*; Kan^R^	This study
*P. putida* PP74 (JPUB_018608)	*P. putida* carrying pRGPspdCas9bad‐*trpG2*; Kan^R^	This study
*P. putida* PP75 (JPUB_018609)	*P. putida* carrying pRGPspdCas9bad‐*trpG3*; Kan^R^	This study
*P. putida* PP76 (JPUB_018610)	*P. putida* carrying pRGPspdCas9bad‐*trpG4*; Kan^R^	This study

^a^ Strain name in bracket corresponds to the part ID assigned to each strain for JBEI public registry.

### Arabinose‐inducible gene expression in *P. putida* KT2440

The *lacI* and *tetR* repressor systems were used in the construction of pRGPDuo1 and pRGPDuo2. Although both *lacI* and *tetR* repressor systems work well in *P. putida* KT2440 (Gautam et al., 2020), concerns were raised in the past about the efficacy of the ATc‐inducible *tetR* repressor system for this organism (Cook *et al*., 2018). In one study, gene expression was enhanced by 38‐fold with the P*_tet_* system (Lee *et al*., 2011), whereas in a similar study, protein expression level could not be improved using P*_tet_* system (Chai *et al*., 2012). These contradictory findings raised doubts regarding reliable gene expression using our duet‐expression vectors pRGPDuo1 and pRGPDuo2, prompting us to look for an alternative substrate‐dependent repressor system. The *araC‐*P*_bad_*‐based repressor system is considered a tightly regulated system in *P. putida* and shown to increase gene expression levels when induced with arabinose (Bi *et al*., 2013; Cook *et al*., 2018). To improve the duet‐expression vectors for reliable gene expression in *P. putida* KT2440, expression vectors pRGPDuo3 and pRGPDuo4 were developed by replacing the *tetR*‐P*_tetR/tetA_* with *araC‐*P*_bad_* while still retaining all other features, including *lacI*‐P*_tac_* (Fig. 1B and C).

The pRGPDuo3‐ and pRGPDuo4‐derived plasmids were electroporated into *P. putida* KT2440 to generate recombinant strains: PP12‐19 (Table 1). The induced strains were grown in the presence of both inducers, and no inducer was added for the uninduced ones (Fig. S1A). To functionally characterize these, plasmid's expression of one reporter gene was confirmed per plasmid. The strains PP12 and PP13 carrying the empty vector(s) were used as control, and the respective uninduced counterpart(s) for the test strain(s) was used to determine the background fluorescence for GFP and RFP (Fig. S1B and C). GFP fluorescence was higher in induced cultures of strains PP14‐17 compared to their uninduced counterparts and the control strains PP12 and PP13 (Fig. 1F). The background fluorescence in strains PP18 and PP19 expressing RFP (not GFP) was comparable to the control strain (Table S4). The higher fluorescence for strains PP14‐17 in the uninduced state compared to control strains PP12 and PP13 indicates leaky GFP expression (Fig. 1F and Table S4). However, GFP expression was tightly controlled in the arabinose‐inducible P*_araC/bad_* than the IPTG‐inducible P*_lacI/tac_* repressor system. Similarly, RFP expression was sixfold to 11‐fold in induced cultures from strains PP18 and PP19, compared to their uninduced counterparts and the cultures from control strains (PP12‐13). The background fluorescence in the cultures from strains PP14‐PP17 expressing GFP (not RFP) was comparable to the uninduced counterpart for strains PP18 and PP19 (Fig. 1G). Contrary to higher background fluorescence in strains expressing GFP, the background fluorescence for RFP was much lower and in the same range as that of the control strains (Table S4). A possible explanation can be lower assay sensitivity for RFP compared to GFP detection assay. To conclude, our results demonstrated the (over)expression of genes in test strains (compared to respective uninduced counterparts) cloned into MCS1 and MCS2, individually controlled by IPTG‐ and arabinose‐controlled promoters P*_tac_* and P*_bad_*, respectively, that in turn, establishes the functional validation of pRGPDuo3 and pRGPDuo4 for gene expression in *P. putida* KT2440.

### Compatibility of pBBR1‐ and pRO1600‐based duet‐expression vectors for gene coexpression in *P. putida* KT2440

**Fig. 2:**
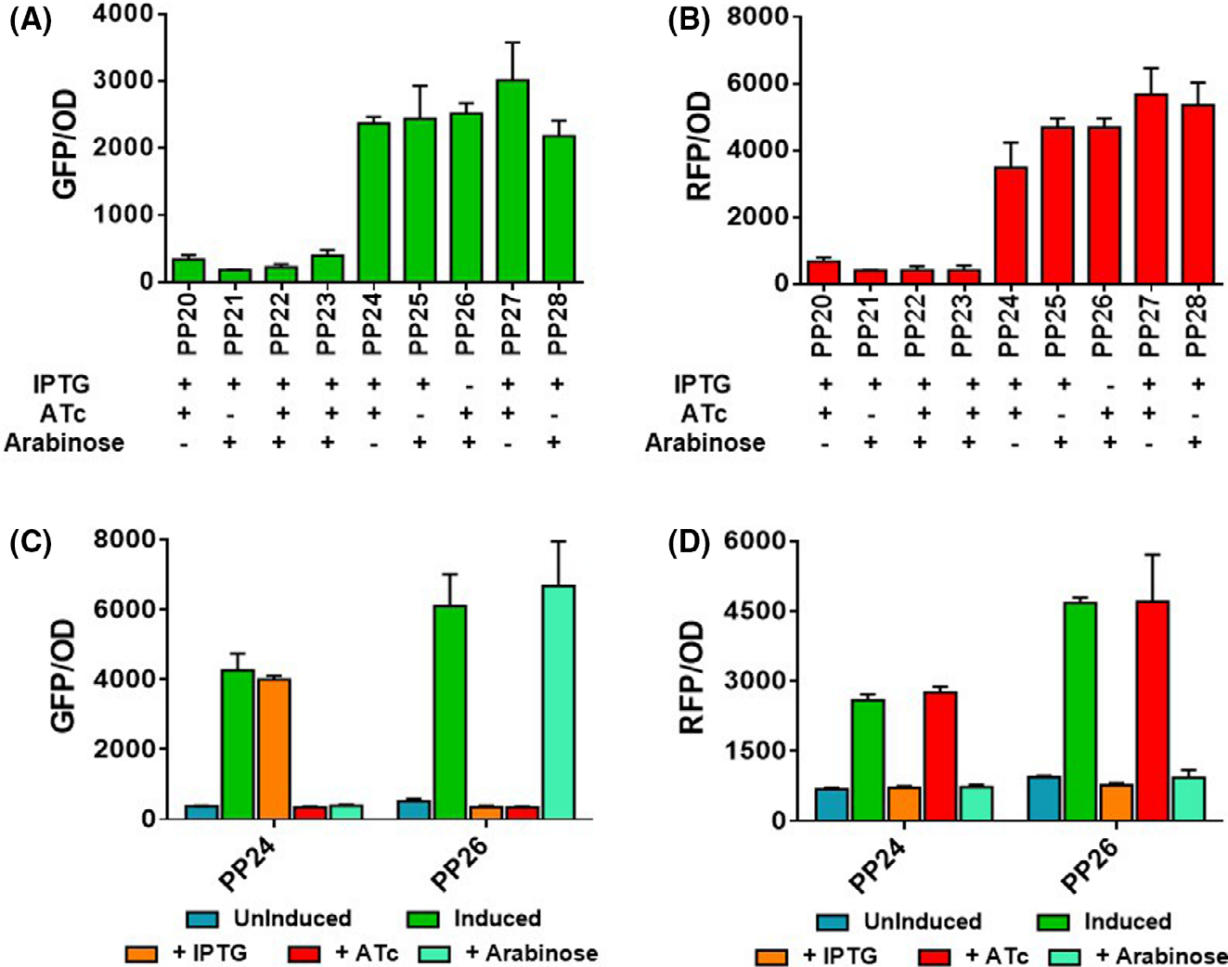
Plasmid compatibility studies of pBBR1‐derived vectors (pRGPDuo1 and pRGPDuo3) with pRO1600‐derived vectors (pRGPDuo2 and pRGPDuo4) in *P. putida* KT2440. The recombinant *P. putida* KT2440 strains were investigated for GFP (A) and RFP (B) fluorescence: PP20 (pRGPDuo1 + pRGPDuo2), PP21 (pRGPDuo3 + pRGPDuo4), PP22 (pRGPDuo1 + pRGPDuo4), PP23 (pRGPDuo2 + pRGPDuo3), PP24 (pRGPDuo1‐sfGFP_tac_ + pRGPDuo2‐RFP_tet_), PP25 (pRGPDuo2‐sfGFP_tac_ + pRGPDuo3‐RFP_bad_), PP26 (pRGPDuo2‐RFP_tet_ + pRGPDuo3‐sfGFP_bad_), PP27 (pRGPDuo2‐RFP_tet_ + pRGPDuo3‐sfGFP_tac_) and PP28 (pRGPDuo3‐sfGFP_tac_ + pRGPDuo4‐RFP_bad_). The (+) sign corresponding to each inducer (below respective strain name) is indicative of the inducer added to express the reporter gene depending on the promoter system (IPTG for P*_tac_*; ATc for P*_tetR/tetA_* and arabinose for P*_araC/bad_*). The GFP (C) and RFP (D) expression levels analysed in the presence of a specific inducer are shown. The annotation indicates the presence or absence of inducers: uninduced (no inducer added); induced (presence of all three inducers: 1 mM IPTG, 1 μg ml^−1^ ATc and 0.2% w/v arabinose), + ATc (only ATc was added), + IPTG (the only IPTG was added) and + arabinose (the only arabinose was added). The graphs consist of relative fluorescence units normalized to OD_600_ during the mid‐exponential phase for each construct. Data represent mean values of triplicate assays from at least two individual cultivations, and error bars represent standard deviations.

The duet vectors created in this study utilize either pBBR1 replicon (pRGPDuo1 and pRGPDuo3) or pRO1600 replicon (pRGPDuo2 and pRGPDuo4) that belongs to the separate incompatibility groups. For stable maintenance of duet plasmids inside a cell when co‐transformed, an appropriate selection marker (kanamycin or gentamicin) was incorporated. The use of compatible vectors can be convenient in situations that require the coexpression of multiple genes. Therefore, we tested the compatibility of the duet vectors in *P. putida* KT2440. For this purpose, the duet plasmids and their derivatives were co‐electroporated into *P. putida* KT2440 to generate recombinant strains PP20‐PP28 (Table 1). Each recombinant strain harboured two plasmids and was grown in the presence of appropriate inducers (1mM IPTG, 1 μg ml^−1^ ATc and 0.2% arabinose), depending on the promoter's combination employed for gene expression. The control strains PP20‐23 harboured a combination of empty vectors. The test strains PP24‐28 also had two plasmids such that the expression of two distinct reporter genes (either sfGFP or RFP) comes from two different plasmids. The GFP fluorescence increased by sixfold to 10‐fold in test strains PP24‐PP28 compared to control strains (Fig. 2A). Similarly, RFP fluorescence increased by eightfold to 13‐fold in test strains compared to the control strains PP20‐23 (Fig. 2B).

Next, fluorescence measurements were performed in the presence of a single inducer to confirm that it does not influence the gene expression from a non‐cognate promoter. The GFP fluorescence for strains PP24 and PP26 was increased only in the presence of respective cognate inducer for PP24 (IPTG) and PP26 (arabinose) (Fig. 2C). In comparison, non‐cognate inducers (such as ATc) did not influence gene expression in any of the strains. Similarly, RFP fluorescence for strains PP24 and PP26 was increased only in the presence of respective cognate inducer(s), in both PP24 and PP26 (ATc). In contrast, non‐cognate inducers (IPTG and arabinose) had RFP levels similar to the uninduced cultures (Fig. 2D). For vector compatibility experiments, the recombinant strains were grown in the presence of two antibiotics to ensure the maintenance of both plasmids. The expression of both sfGFP and RFP in the same strain (PP24‐28) confirmed the functionality and compatibility of duet vectors. In summary, we have shown the coexpression of two independently regulated reporter genes (sfGFP and RFP) using a combination of two plasmids, which in turn establishes the compatibility of pBBR1‐ and pRO1600‐derived dual‐inducible duet‐expression plasmids in *P. putida* KT2440.

### Influence of inducer(s) concentration on GFP and RFP expression

**Fig. 3:**
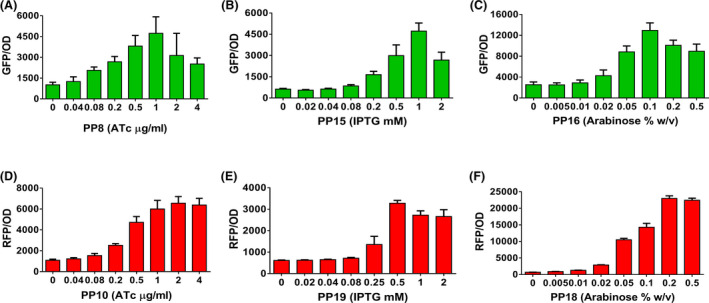
Titration of reporter gene expression using duet‐expression vectors in *P. putida* KT2440. (A, B, C) GFP levels of recombinant strains PP8 (pRGPDuo1‐sfGFP_tet_), PP15 (pRGPDuo3‐sfGFP_tac_) and PP16 (pRGPDuo4‐sfGFP_bad_) upon induction with serial concentrations of specific inducer, namely ATc (ranging from 0 to 4 μg ml^−1^), IPTG (ranging from 0 to 2 mM) and arabinose (ranging from 0 to 0.5% w/v). (D, E, F) RFP levels of recombinant strains PP10 (pRGPDuo1‐RFP_tet_), PP19 (pRGPDuo4‐RFP_tac_) and PP18 (pRGPDuo4‐RFP_bad_) upon induction with serial concentrations of specific inducer. Data represent mean values of triplicate assays from at least two individual cultivations, and error bars represent standard deviations.

The tunability of an expression plasmid is a crucial feature during heterologous protein expression. Next, we investigated the dose‐response of GFP and RFP expression in these plasmids. For this purpose, GFP and RFP expression was analysed at varying ATc (ranging from 0 to 4 μg ml^−1^), IPTG (ranging from 0 to 2 mM) and arabinose (ranging from 0 to 0.5% w/v) concentrations. GFP levels were quantified in strains PP8 (varying ATc and fixed 1mM IPTG), PP15 (varying IPTG and fixed 0.2% arabinose) and PP16 (varying arabinose and fixed 1mM IPTG). For each of these strains, GFP levels were modulated in a dose‐dependent manner. For example, when the strain PP8 was induced with 0.04 μg ml^−1^ ATc, the GFP levels (1253 ± 152 GFP/OD) were comparable to the levels (1015 ± 82 GFP/OD) in the absence of ATc. Maximum GFP expression (4731 ± 533 GFP/OD) at 1 μg ml^−1^ was almost fourfold higher than in the absence of inducer (Fig. 3A). The results show that the P*_tetR/tetA_* promoter is responsive to low ATc concentration for gene expression, and expression level reached saturation at higher concentration of inducer (> 1 μg ml^−1^) (Fig. 3A). Similarly, GFP fluorescence increased in strains PP15 and PP16, from 629 ± 20 GFP/OD_600_ in the absence of inducer to 4723 ± 253 GFP/OD_600_ in the presence of 1mM IPTG and from 2542 ± 243 GFP/OD_600_ in the absence of inducer to 12 941 ± 645 GFP/OD_600_ in the presence of 0.1% arabinose respectively (Fig. 3B and C). Again, a saturation of gene expression was observed for both strains, and a high concentration of IPTG (> 1mM) and arabinose (> 0.1%) failed to increase the fluorescence levels beyond threshold relative fluorescence units (RFU) (Fig. 3B and C). Moreover, the same experiments were performed to quantify the RFP levels in strains PP10 (varying ATc and fixed 1mM IPTG), PP19 (varying IPTG and fixed 0.2% arabinose) and PP18 (varying arabinose and fixed 1mM IPTG). The observations were quite similar to what we have reported for GFP fluorescence. For instance, no apparent difference in RFP fluorescence was observed for strain PP10 in the presence of ATc concentrations ranging from 0 to 0.08 μg ml^−1^ and linear increase in RFP was observed in the presence of ATc concentrations ranging from 0.2 to 1 μg ml^−1^ that reaches to saturation at very high ATc concentrations (> 1 μg ml^−1^) (Fig. 3D). The lower IPTG (ranging from 0 to 0.08 mM) and arabinose (ranging from 0 to 0.02%) concentrations did not increase the RFP levels in strains PP19 and PP18 respectively (Fig. 3E and F). Higher concentrations of IPTG (ranging from 0.25 mM to 2 mM) and arabinose (ranging from 0.02% to 0.5%) increased the RFP levels nearly fivefold and 30‐fold, respectively, compared to the one without inducer (Fig. 3E and F). In conclusion, the constructed dual‐inducible vectors allow the titrable expression of target genes and can be used to fine‐tune the expression of critical genes in biosynthetic pathways.

### Application of dual‐promoter system for CRISPR interference‐mediated gene repression in *P. putida* KT2440

**Fig. 4:**
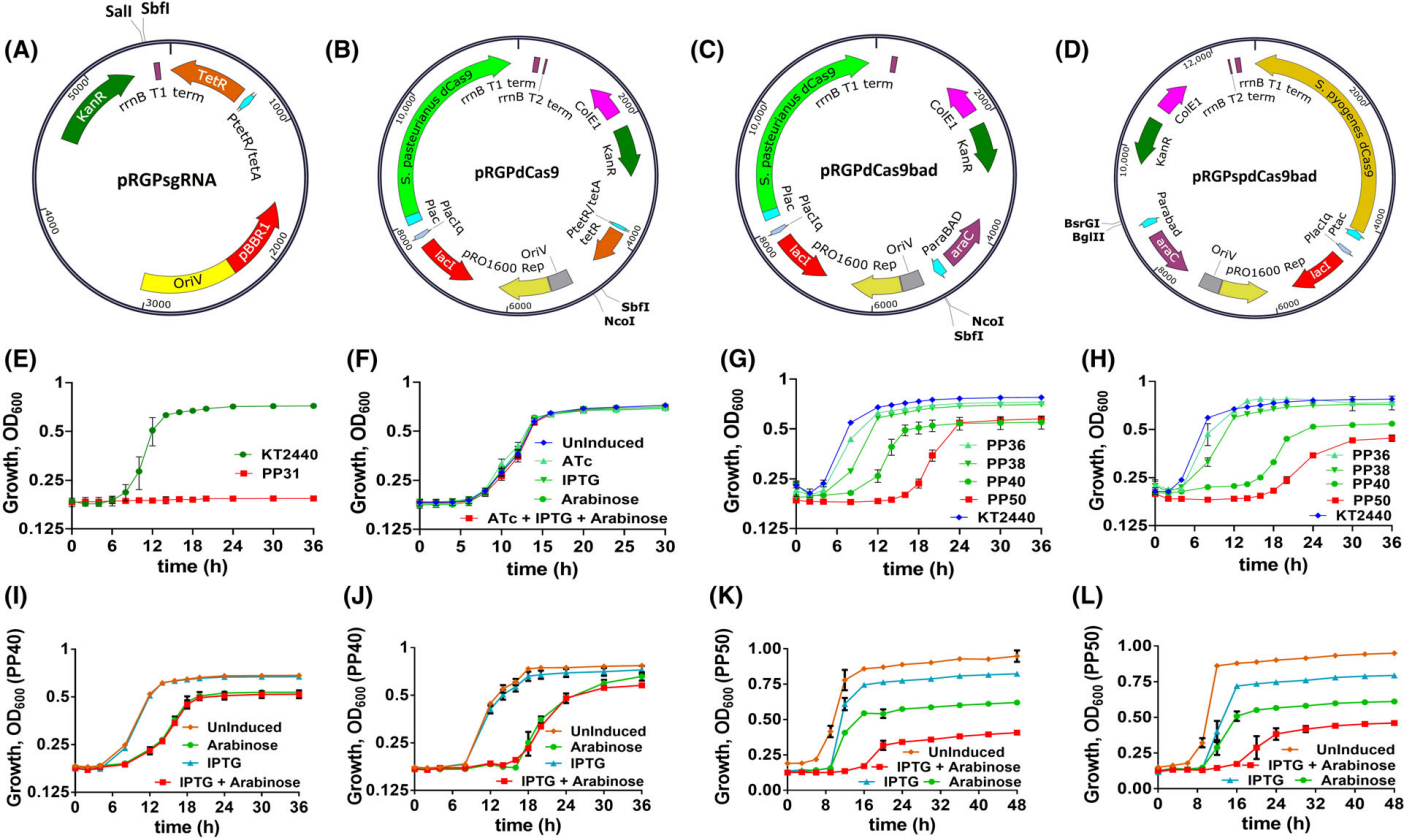
Vector maps of sgRNA vector pRGPsgRNA (A) and dual‐inducible CRISPRi vectors pRGPdCas9 (B), pRGPdCas9bad (C) and pRGPspdCas9bad (D). Growth analysis of *P. putida* KT2440 and its derivative strains was performed in M9 minimal media with glucose as C‐source. Comparison of growth for *P. putida* KT2440 with deletion mutant strain PP31 (deletion of *aceEF* operon) (E). Growth analysis in the presence of different inducers (1 mM IPTG, 1 μg ml^−1^ ATc and 0.2% w/v arabinose) when precultures were induced (F). Growth curves of strains PP36 (pRGPsgRNA‐*aceE*), PP38 (pRGPdCas9‐*aceE*), PP40 (pRGPdCas9bad‐*aceE*) and PP50 (carrying pRGPspdCas9bad‐*aceE1*), compared to wild‐type strain *P. putida* KT2440 using CRISPRi, when precultures were not induced (G), and when precultures were induced (H). Leakiness of *S. pasteurianus* dCas9‐based CRISPRi system (pRGPdCas9bad) and its effect on growth when precultures were not induced (I) and when precultures were induced (J). Leakiness of *S. pyogenes* spdCas9‐based CRISPRi system (pRGPspdCas9bad) and its effect on growth when precultures were not induced (K) and when precultures were induced (L). The annotation indicates the presence or absence of inducers: uninduced (no inducer added); induced (presence of both inducers: 1 mM IPTG and 0.2% w/v arabinose), + IPTG (the only IPTG was added) and + arabinose (the only arabinose was added). Each graph represents the mean values of biological triplicates from at least two individual cultivations, and error bars represent standard deviations.

The dual‐inducible vectors were designed to bring tighter control over gene expression. To further demonstrate our duet‐expression vector's ability for metabolic engineering purposes, we employed these vectors to develop a robust CRISPR interference system for targeted gene repression in *P. putida* KT2440. This study utilized both type II dCas9 homologs from *S. pasteurianus* (dCas9) and *S. pyogenes* (spdCas9) to demonstrate gene repression. The vectors pRGPdCas9 (Fig. 4B) and pRGPdCas9bad (Fig. 4C) were constructed by cloning *dCas9* into pRGPDuo2 under the control of IPTG‐inducible P*_lac_* promoter. *spdCas9* was cloned into pRGPDuo4 under the control of IPTG‐inducible P*_tac_* promoter to construct pRGPspdCas9bad (Fig. 4D). To select the appropriate induction system for controlling the sgRNA expression, we created two variants of dCas9‐based CRISPRi vectors, pRGPdCas9 (ATc‐inducible sgRNA expression) and pRGPdCas9bad (arabinose‐inducible sgRNA expression). To compare the plasmid‐based CRISPRi vectors' performance with dCas9 genomic integration‐based CRISPRi system (Tan *et al*. (2018), sgRNA plasmid pRGPsgRNA was constructed for ATc‐inducible sgRNA expression. To demonstrate the dual‐inducible CRISPRi system's functionality, we targeted *aceE* (*PP_0339*) gene, which encodes for subunits of pyruvate dehydrogenase, an essential gene for growth on glucose (Wirth *et al*., 2020). Therefore, repression could be assessed directly via growth assay. A knockout strain PP31 (deletion of *PP_0338‐PP_0339*) was constructed, and its growth phenotype was compared to wild‐type *P. putida* KT2440. As expected, the deletion of *aceEF* prevented *P. putida* growth in minimal media with glucose as the sole carbon source (Fig. 4E). However, the growth was not affected when the strain PP31 was grown in minimal medium supplemented with *p*‐coumaric acid (Fig. S5B). Therefore, *aceE* encoding for pyruvate dehydrogenase subunit E1 was chosen to be the target for demonstrating the functionality of CRISPRi vectors and following *aceE* targeting CRISPRi vectors were constructed: pRGPsgRNA‐*aceE*, pRGPdCas9‐*aceE*, pRGPdCas9bad‐*aceE* and pRGPspdCas9bad‐*aceE1*. The constructed plasmids were transformed in *P. putida* KT2440 to create recombinant strains PP35‐PP40, PP49 and PP50 (Table 1), and growth was analysed in minimal media with glucose (0.5%) with respective inducers. The presence of the inducer(s) did not affect *P. putida* KT2440 growth (Fig. 4F and S4A). The growth of control strain *P. putida* KT2440 was compared with the test strains PP36 (IPTG‐ and ATc‐inducible), PP38 (IPTG‐ and ATc‐inducible), PP40 (IPTG‐ and arabinose‐inducible) and PP50 (IPTG‐ and arabinose‐inducible) under two culture conditions, one where precultures were not induced and other when precultures were induced. The growth remained unaffected for strains PP36 and PP38 compared to *P. putida* KT2440 when precultures were not induced (Fig. 4G). However, the inhibitory effect on growth was observed for strains PP40 and PP50 compared to control strain *P. putida* KT2440. Similarly, growth was not affected for PP36 and PP38 than *P. putida* KT2440 when precultures were induced (Fig. 4H). But, stronger growth impairment was observed for strains PP40 and PP50 when precultures were induced compared to *P. putida* KT2440 (Fig. 4H). The growth inhibition for PP40 was even more potent than its counterpart when precultures were not induced, but the growth difference was not comparable for strain PP50 under both conditions. Moreover, the strains that expressed dCas9 only grew similar to *P. putida* KT2440, indicating no effect of dCas9 alone on growth (Fig. S4B and C). The growth of uninduced PP40 was not affected which indicates the tight regulation of gene repression using CRISPRi (Fig. S4D and E). Interestingly, the growth of PP50 remained unaffected when grown in minimal medium supplemented with *p*‐coumaric acid (similar to PP31), which substantiates the hypothesis that the growth defect in strain PP50 is due to *aceE* downregulation by CRISPRi (Fig. S5D). Furthermore, we investigated the leakiness of our dual‐inducible CRISPRi systems by analysing the growth behaviour of strains PP40 and PP50 in the presence of a different combination of inducers (IPTG and arabinose). The difference in growth behaviour was visible for uninduced and induced counterparts for PP40 and PP50 strains (Fig. 4I–L). Interestingly, using IPTG alone, no growth defect was observed for PP40, indicating tighter control over arabinose‐controlled sgRNA expression. The PP40 strain grew with a reduced growth rate similar to its induced counterpart in the presence of arabinose alone, demonstrating leaky expression of the IPTG‐inducible dCas9 expression (Fig. 4I and J). The leakiness of IPTG‐inducible P*_lac_* for CRISPRi studies has also been reported in previous studies (Tan *et al*., 2018). However, using IPTG alone, a slight growth defect was observed for PP50 that further increased in the presence of arabinose compared to their uninduced counterpart (Fig. 4K and L). Interestingly, the most substantial growth impairment was observed for the induced (IPTG + arabinose) PP50 counterpart, indicating a cumulative effect of both inducible systems on CRISPRi‐based gene repression (Fig. 4K and L). Therefore, the induction of sgRNA alone is sufficient to cause a growth defect using *S. pasteurianus* dCas9‐based CRISPRi. In contrast, the combined use of IPTG and arabinose provided the most robust gene repression using *S. pyogenes* spdCas9‐based CRISPRi. In conclusion, the results indicate that the fully inducible CRISPRi vectors pRGPdCas9bad and pRGPspdCas9bad can be used to knockdown target genes in *P. putida* KT2440.

### CRISPR interference‐mediated repression of conditionally essential genes in *P. putida* KT2440

To demonstrate the robustness of developed CRISPRi vectors (pRGPdCas9bad and pRGPspdCas9bad), eight target genes were selected to test CRISPRi‐mediated gene repression, namely acetylglutamate kinase (*argB*), argininosuccinate lyase (*argH*), 2‐keto‐3‐deoxy‐6‐phosphogluconate aldolase (*eda*), phosphogluconate dehydratase (*edd*), cell division protein (*ftsZ*), chorismate mutase (*pheA*), orotidine‐5’‐phosphate decarboxylase (*pyrF*) and anthranilate synthase component 2 (*trpG*). The selected targets are conditionally essential genes for glucose growth in minimal medium (Molina‐Henares *et al*., 2010; Kuepper *et al*., 2015). For gene targeting using pRGPdCas9bad, eight sgRNAs were designed: four sgRNAs (*argB*, *edd*, *pyrF and trpG*) were targeted in close proximity to the start codon (+10 to +40) on NT strand at the 3' end, three sgRNAs (*argH*, *ftsZ and pheA*) were targeted inside the open reading frame (ORF) of a gene (between +50 to +150) on NT strand at the 3' end and one sgRNA (*eda*) targeted upstream of the start codon (−20 to −30) at 5' end (Table S5). Two PAM sites (5'‐NNGTGA‐3' and 5'‐NNGCGA‐3') were used for sgRNA design (Tan *et al*., 2018) (Table S5). The pRGPdCas9bad‐derived plasmids were transformed in *P. putida* KT2440 to create test strains PP41‐PP48 (Table 1). Similarly, guide RNAs were designed to target the same eight genes using pRGPspdCas9bad, and the following test strains were created: PP54, PP56, PP59‐60, PP64‐65, PP69 and PP73 (Table 1).

**Fig. 5:**
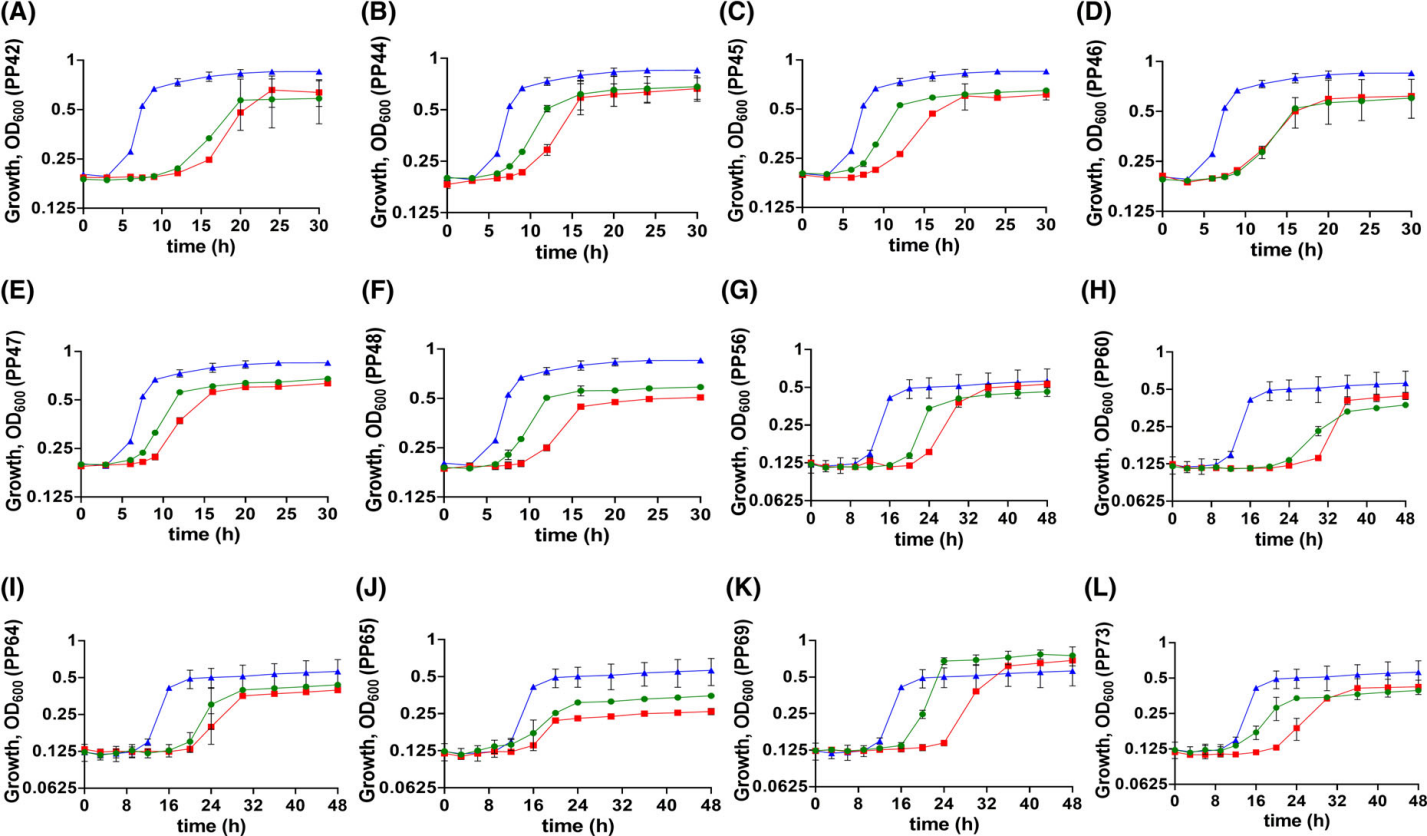
Gene knockdown of essential genes in *P. putida* KT2440 using *S. pasteurianus* dCas9‐based CRISPRi system (A–F) and *S. pyogenes* spdCas9‐based CRISPRi system (G–L). sgRNAs were designed to downregulate the expression of essential genes, namely *argH* (A, G), *edd* (B, H), *ftsZ* (C, I), *pheA* (D, J), *pyrF* (E, K) and *trpG* (F, L). Growth phenotype was assessed for the strains PP42 (pRGPdCas9bad‐*argH*), PP44 (pRGPdCas9bad‐*edd*), PP45 (pRGPdCas9bad‐*ftsZ*), PP46 (pRGPdCas9bad‐*pheA*), PP47 (pRGPdCas9bad‐*pyrF*), PP48 (pRGPdCas9bad‐*trpG*), PP56 (pRGPspdCas9bad‐*argH1*), PP60 (pRGPspdCas9bad‐*edd1*), PP64 (pRGPspdCas9bad‐*ftsZ1*), PP65 (pRGPspdCas9bad‐*pheA1*), PP69 (pRGPspdCas9bad‐*pyrF1*) and PP73 (pRGPspdCas9bad‐*trpG1*) and compared to the respective control strain PP39 (pRGPdCas9bad) or PP49 (pRGPspdCas9bad). In each graph, control strain PP39 or PP49 is represented as a blue triangle. The green circle in each graph represents the respective strain when precultures were not induced. The red square in each graph represents the respective strain when precultures were induced. Each graph represents the mean values of biological triplicates from at least two individual cultivations, and error bars represent standard deviations.

For phenotypic characterization of recombinant CRISPRi strains, the growth experiments were performed in M9 glucose (0.5% w/v) under two culture conditions: i) precultures were not induced and ii) precultures were induced; PP39 and PP49 carrying empty CRISPRi plasmids were used as control strains. Using the *S. pasteurianus* dCas9‐based CRISPRi system, growth was repressed for seven test strains upon induction: namely PP41 (Fig. S7A), PP42 (Fig. 5A), PP44 (Fig. 5B), PP45 (Fig. 5C), PP46 (Fig. 5D), PP47 (Fig. 5E) and PP48 (Fig. 5F) compared to the control strain PP39. Even though these strains grew with reduced growth rates, the final OD_600nm_ was comparable to *P. putida* KT2400. However, the growth of PP43 was reduced marginally compared to control strain PP39 (Fig. S7B). Both PAM sites (5'‐NNGTGA‐3' and 5'‐NNGCGA‐3') worked well and provided robust gene repression with pRGPdCas9bad. The measurement of glucose consumption in recombinant strains corroborated the results obtained for the growth data. The PP39, PP43 and control *P. putida* KT2440 consumed the entire glucose within the first 12 h of growth (Fig. S8G and H). On the contrary, glucose consumption was slow in strains PP42, PP44 and PP46 compared to *P. putida* KT2440 where the strains did not metabolize all the glucose within the first 12 h of growth (Fig. S8G and H). The growth consumption data indicate that test strains with growth phenotype consumed glucose slowly compared to control strain. Similarly, growth was affected for all strains (except PP54; Fig. S7C), namely PP56 (Fig. 5G), PP59 (Fig. S7D), PP60 (Fig. 5H), PP64 (Fig. 5I), PP65 (Fig. 5J), PP69 (Fig. 5K) and PP73 (Fig. 5L) compared to the control strain PP49 upon induction using *S. pyogenes* spdCas9‐based CRISPRi system. The control strain growth was comparable to the uninduced counterpart indicating tighter control over gene repression using a dual‐promoter‐based CRISPRi system. The growth phenotype was more robust when precultures were induced in *S. pasteurianus* dCas9‐based gene repression. However, the trend was not consistent for *S. pyogenes* spdCas9‐based gene repression, and growth curves were comparable irrespective of whether the precultures were induced or not.

**Fig. 6:**
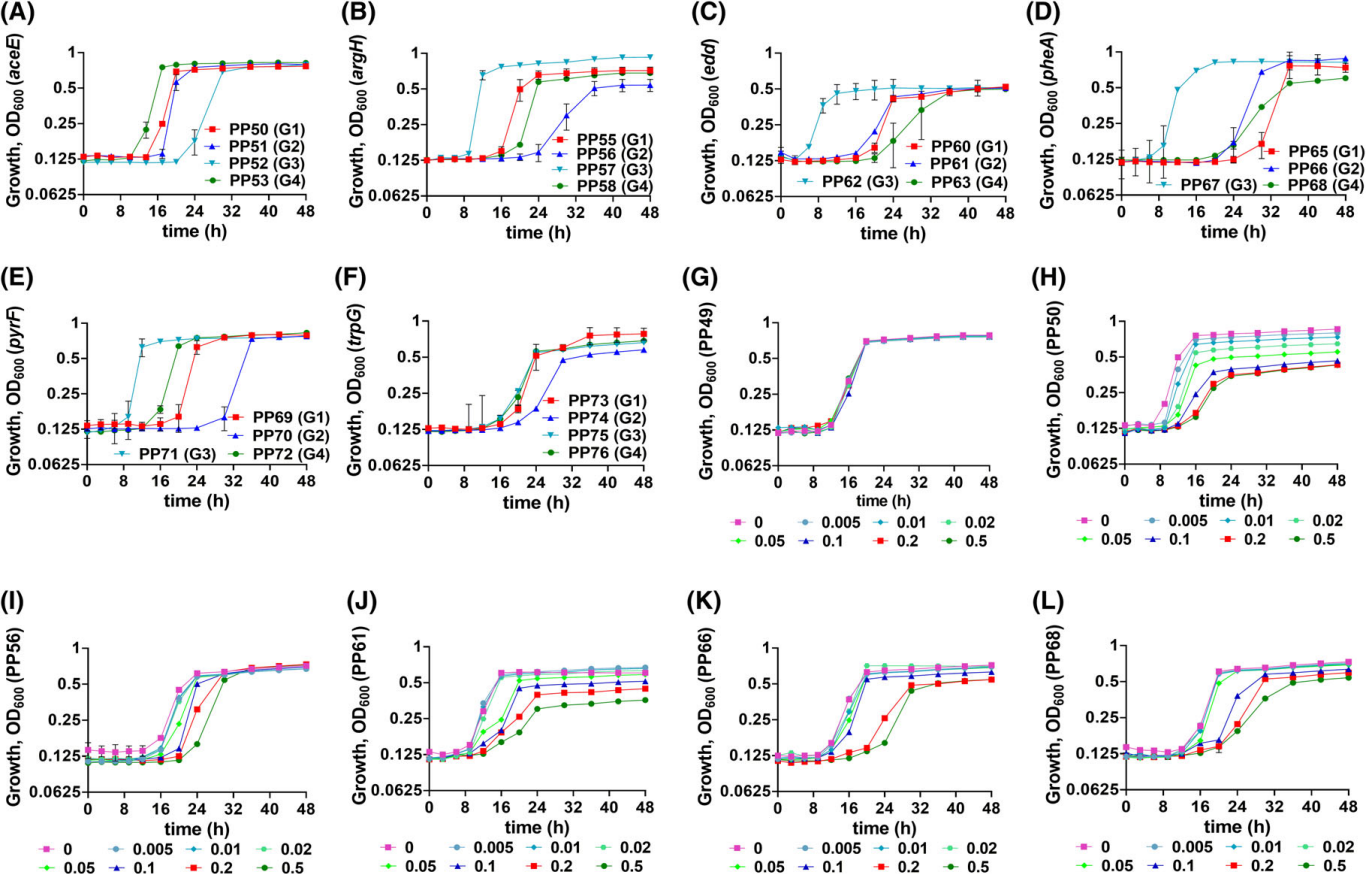
Evaluation of the effect of sgRNA location on knockdown efficiency using *S. pyogenes* spdCas9‐based CRISPRi system. Four sgRNAs‐G(1‐4) each targeting a different location relative to start codon (in the same gene) were designed to target six genes *aceE* (A), *argH* (B), *edd* (C), *pheA* (D), *pyrF* (E) and *trpG* (F). The CRISPRi‐mediated (pRGPspdCas9bad based) tunable repression of essential genes in *P. putida* KT2440 was analysed. The expression of spdCas9 from *Streptococcus pyogenes* is under *P_tac_* control (IPTG‐inducible), and the strong pBAD promoter (arabinose‐inducible) controls sgRNA expression. Growth of recombinant *P. putida* strains PP49 (G), PP50 (H), PP56 (I), PP61 (J), PP66 (K) and PP68 (L) was investigated in the presence of different concentrations of arabinose (ranging from 0 to 0.5% w/v) and a constant IPTG (1 mM) amount. For this set of experiments, precultures were not induced. For the description of strains, refer to Table 1. Each graph represents the mean values of biological triplicates from at least two individual cultivations, and error bars represent standard deviations.

Next, we evaluated the effect of sgRNA position on gene repression efficiency using SpdCas9‐based CRISPRi system. For this purpose, four different sgRNAs‐G(1‐4) were designed to target six genes (*aceE*, *argH*, *edd*, *pheA*, *pyrF* and *trpG*) targeting the non‐template (NT) strand, whereby G(1‐4) stands for different sgRNAs targeting the same gene depending on the location (−50 to +250) (Table S5). Three sgRNAs (G1, G3 and G4) targeted the coding region of genes downstream of the start codon at 3’‐end and one sgRNA (G2) targeted upstream of start codon at 5’‐end (Table S5). For strain‐description, refer to Table 1 and supplementary provided (Table S1). The results demonstrated that different sgRNAs targeting the same gene affected the growth to a different extent (Fig. 6A–F). Out of six genes targeted, three genes (*argH*, *pyrF* and *trpG*) targeting the location upstream of the start codon (G2) were more effective (Fig. 6B and E–F), and results corroborate previous studies (Tan *et al*., 2018; Tian *et al*., 2019). However, for the other three targets, results were not consistent, and distinct sgRNA location(s) provided the most robust repression efficiency: G3 for *aceE* (Fig. 6A), G4 for *edd* (Fig. 6C) and G1 for *pheA* (Fig. 6D). Collectively, most of the designed sgRNAs showed high gene knockdown efficiency. To summarize the results, we have successfully demonstrated duet‐expression vector's utility to create a robust and reliable gene repression CRISPRi system.

Finally, we sought to explore if our CRISPRi system that combines the use of two different inducible promoter systems could be fine‐tuned to achieve precise control over transcriptional repression. We noticed that IPTG‐inducible *P_tac_* (reporter gene expression and spdCas9 expression) and P*_lac_* (dCas9 expression) repressor systems were leaky (Fig. S9). However, arabinose‐controlled P*_araC/bad_* repression machinery tightly regulates genes' expression, including sgRNA transcription (Fig. S6). To demonstrate the tunable gene repression, growth experiments were performed using different arabinose concentrations (ranging from 0% and 0.5%) and a fixed IPTG concentration (1mM). The growth behaviour of *S. pyogenes*‐based CRISPRi vector was analysed for six recombinant strains, PP49 (Fig. 6G), PP50 (Fig. 6H), PP56 (Fig. 6I), PP61 (Fig. 6J), PP66 (Fig. 6K) and PP68 (Fig. 6L) in the presence of varying arabinose concentrations, and the precultures were not induced. The gradual increase in arabinose concentration (0.02 to 0.5% w/v) leads to differential repression of genes with maximum gene repression at higher arabinose concentrations (0.1, 0.2, 0.5%). The growth was comparable when arabinose concentration was low (0.005, 0.01%) compared to the uninduced state. Similar growth trends were observed for *S. pasteurianus*‐based CRISPRi‐targeted strains PP41 (Fig. S8A), PP42 (Fig. S8B), PP44 (Fig. S8C), PP45 (Fig. S8D), PP46 (Fig. S8E) and PP48 (Fig. S8F), and strongest gene repression was observed for higher arabinose concentration and a negligible growth phenotype was observed at lower concentrations compared to their uninduced counterparts. The results were not consistent when similar experiments were performed with varying IPTG concentrations (Fig. S9A–F). To summarize our findings, the CRISPRi system is titrable for inducer arabinose. It can influence growth phenotype (especially in the case of essential genes) by altering the concentration of arabinose that controls the sgRNA expression.

## Discussion

In this study, we report the development of a series of flexible dual‐inducible duet‐expression vectors. Two of these vectors (pRGPDuo1 and pRGPDuo3) utilized the pBBR1 replicon, known to stably replicate in gram‐negative bacteria (Prior *et al*., 2010; Calero *et al*., 2016). The vector pRGPDuo4 (and also pRGPDuo2) was derived using pRO1600, a replication origin that is known to replicate in number of prokaryotic species including *Klebsiella pneumoniae* (Olsen *et al*., 1982), *Burkholderia* spp (Choi *et al*., 2008), *Salmonella* spp (Nakata, 2017), *P. aeruginosa* (Olsen *et al*., 1982), *P. fluorescens* (Nakata, 2017) and *P. putida* (Gauttam *et al*., 2020). The characteristic of our duet plasmids to replicate in a wide range of hosts makes them useful broad‐host‐range shuttle vectors that can also be used in other microorganisms in addition to *Pseudomonas* for the biotechnological applications. All expression plasmids constructed in this study functions in both *P. putida* KT2440 and *E. coli*, which provides an advantage of characterizing genes of interest (GOI) simultaneously in both the organisms to implement metabolic engineering approaches without undergoing additional cloning works and calibration for gene expression.

Here, we tested three different inducible promoter systems (*lacI*/P*_tac_*, *tetR*/P*_tetR/tetA_* and *araC*/P*_bad_*) and combined the use of two of them in the same construct to allow tunable expression of two different genes. The leakiness for gene expression was observed for all three promoter systems, with IPTG‐inducible *lacI*/P*_tac_* being the leakiest one followed by ATc‐inducible *tetR*/P*_tetR/tetA_* and arabinose‐inducible *araC*/P*_bad_* (P*_tac_* > P*_tetR/tetA_* > P*_bad_*). The dual‐inducible feature of duet vectors provides better control over gene expression (Tolia and Joshua‐Tor, 2006; Gauttam *et al*., 2019a). An appropriate promoter–repressor system plays a crucial role in designing metabolic engineering strategies in situations where the product of some genes is required in a lower amount, and a higher product is desired for the other genes (Cook *et al*., 2018). In such cases, a more potent promoter is used to express the gene(s) whose product is required in a high amount or vice versa (Mueller *et al*., 2018). The quantification of fluorescence signals revealed the promoter's strength for gene expression in the following order: P*_bad_* > P*_tac_*_ _> P*_tetR/tetA_*. The results substantiate the previous findings that *araC*/P*_bad_* is the most robust one for gene expression due to stronger gene expression and tighter control in the uninduced state among all the three promoter systems tested in this study (Bi *et al*., 2013; Graf and Altenbuchner, 2013). The gene expression was titrable for all three promoter systems. Moreover, for specific purposes, the appropriate choice of two compatible duet vectors allows the user to individually regulate the expression of at least three genes in the same cell. Further, the duet vectors can be modified easily to extend to a fourth repressor‐based promoter system and also to test different repressor systems to create a platform strain for optimized recombinant protein production in *P. putida* KT2440.

To demonstrate that the scope of the duet plasmids is not limited to gene (over)expression, the vectors were modified to create a single‐plasmid‐based CRISPRi system for gene repression in *P. putida* KT2440. Tan *et al*. (2018) showed CRISPRi technology's applicability (*S. pasteurianus* dCas9 based) for gene repression in *P. putida*, using a two‐plasmid‐based integrative CRISPRi system. However, the system was found to be leaky and restricts the system's use for targeting essential genes. Moreover, the use of two plasmids limits the scope for further engineering towards developing a platform strain (Witthoff *et al*., 2015). The genomic integration‐based CRISPRi system limits the simultaneous examination of gene(s) knockdown effect in the genetically distinct background. These limitations can be overcome using a single‐plasmid‐based CRISPRi system (Gauttam *et al*., 2019b). Recently, two independent groups demonstrated using a single‐plasmid‐based CRISPRi vector to repress genes in *P. putida* (Batianis *et al*., 2020; Kim *et al*., 2020). Both studies utilized dCas9 from *Streptococcus pyogenes* (SpdCas9) whose expression was controlled using an inducible promoter system and which used constitutive expression of sgRNA. Here, we compared the advantages of using *S. pasteurianus* dCas9 and *S. pyogenes* spdCas9 in *P. putida* KT2440. The expression of dCas9 (*S. pasteurianus*) alone did not affect the *P. putida* KT2440 growth, whereas a longer lag phase was observed for strains expressing spdCas9 (*S*. *pyogenes*), indicating tat expression of spdCas9 has some effect on *P. putida* KT2440 growth. The intent behind developing a fully inducible CRISPRi system was to reduce the overall leakiness of CRISPRi machinery. Combining two different inducible systems can cumulatively provide enhanced control over CRISPRi machinery for gene regulation studies by overcoming the limitations of one inducer by the other one. For *S. pasteurianus* dCas9‐based constructs, growth was affected when strains were grown in the presence of arabinose only, whereas no effect on growth was observed when strains were grown in IPTG only. More potent growth inhibition was observed for cultures when precultures were induced (with IPTG and arabinose), indicating the importance of induction timing for efficient repression. For *S. pyogenes* spdCas9‐based constructs, growth was slightly affected when strains were grown in the presence of IPTG only, and the stronger repression was observed when only arabinose was added. Interestingly, the cumulative effect of both inducible systems (P*_tac_* and P*_araC/bad_*) proved to be more effective in gene repression, and growth impairment was maximized when induced with IPTG and arabinose both compared to the condition when only one of the inducers was added. In the case of spdCas9‐based gene repression, the induction of preculture does not seem to improve its gene repression efficiency and the growth impairment was comparable under both conditions tested. Also, the results indicate leaky expression of dCas9 consistent with previous findings by Tan *et al*. (2018), whereas sgRNA expression is tightly controlled by *araC*/P*_bad_* repressor system. Therefore, to improve the CRISPRi system and reduce the leaky expression of spdCas9, different inducible systems, instead of those tested in the present study, may be employed in the future.

The efficiency of the CRISPRi system for targeted inhibition depends on the binding site of the sgRNAs (Radzisheuskaya *et al*., 2016). A major advantage in using *S. pyogenes* spdCas9 is that the PAM sequence for spdCas9 (NGG) is shorter; therefore, these PAM sites are present in abundance in *P. putida* genome compared to the longer PAM sequences for *S. pasteurianus* dCas9 (NNGTGA and NNGCGA). To compare the CRISPRi‐mediated gene repression efficiency, four sgRNAs targeting different location in same gene were tested and growth phenotype was observed for almost every strain that we analysed. In most cases, repression was stronger when sgRNAs binding sites were located in close proximity to the transcription start site (TSS) to prevent transcription initiation. Therefore, it is safe to target a region within the ORF that blocks transcriptional elongation especially if the location of the promoter is unknown. Moreover, the dual‐inducible CRISPRi platform is tunable, and gene repression can be modulated using different arabinose concentrations. Overall, the plasmid‐based CRISPRi system is a reliable and robust tool for genomic manipulation and reduces the overall leaky expression of CRISPRi machinery for gene repression.

In conclusion, we have developed and functionally characterized broad‐host‐range expression plasmids for *P. putida* KT2440 that can also be adapted for other microorganisms. The plasmids are an important addition to the existing repertoire of inducible promoter systems and should contribute to further development of *P. putida* research and in producing valuable bioproducts.

## Experimental procedures

### Bacterial strains, plasmids and culture conditions

*Escherichia coli* DH5α (Hanahan, 1983) was employed as the host for routine DNA manipulations such as gene cloning and plasmid isolation. *Pseudomonas putida* KT2440 was used as the host strain for expression experiments. All bacterial strains and plasmids used in this study are described in Table 1 and Table S1 respectively. *E. coli* strains were routinely propagated in LB broth (tryptone 10 g l^−1^, yeast extract 5 g l^−1^, NaCl 2.5 g l^−1^) at 37°C on a shaker incubator at 200 rpm. For expression experiments, *P. putida* (precultures only) were grown in LB media at 30°C at 200 rpm in a shaking incubator. For plasmid selection or stable maintenance of compatible plasmids, cultivation medium was always supplemented with appropriate antibiotics, namely kanamycin (50 µg ml^−1^) and gentamicin (30 µg ml^−1^). To induce the gene expression or subsequent protein production, inducers were added in an appropriate amount. Unless otherwise stated, the following concentrations were used: for IPTG (1mM), ATc (1 μg ml^−1^) and arabinose (0.2% w/v). For expression experiments in *P. putida* KT2440, a single colony was picked from a freshly prepared agar plate to inoculate 5 ml of LB medium (first preculture) and grown aerobically (overnight) at 30°C with 200 rpm. This first preculture was used as inoculum for the second preculture, which contained M9 minimal media (6 g l^‐1^ (Na_2_HPO_4_), 3 g l^‐1^ KH_2_PO_4_, 1.4 g l^‐1^ (NH_4_)_2_SO_4_, 0.5 g l^‐1^ NaCl, 0.2 g l^‐1^ MgSO_4_.7H_2_O) supplemented with trace element solution purchased from Teknova (1 ml l^‐1^) and carbon source glucose (0.5%). All recombinant *P. putida* KT2440 strains were grown two times in the M9 medium before inoculating into the main culture (M9 medium) for growth kinetics (and fluorescence experiments). The cultivation experiments were performed in triplicate, and growth was determined spectrophotometrically by measuring the absorbance at 600 nm (OD_600_). All recombinant strains and plasmids used in this study have been deposited in the public instance of the JBEI Registry (http://public‐registry.jbei.org/).

### DNA manipulation and plasmid construction

Restriction enzymes, Fast alkaline phosphatase (FastAP), T4 DNA Ligase and DNA Ladder were ordered from Thermo Fisher Scientific (Waltham MA, USA) and used as per instructions from the manufacturer. DNA isolation, ligation, gel electrophoresis, gene cloning, *E. coli* competent cell preparation and transformation were performed using standard molecular biology techniques (Green and Sambrook, 2012). The sequences of the oligonucleotides and synthetic sgRNAs used in this study are listed in Tables S2 and S3 and ordered from Integrated DNA Technologies (IDT, San Diego, CA, USA). Gene fragments for cloning were amplified using proofreading enzyme Q5 High‐Fidelity DNA polymerase (New England Biolabs, Ipswich, MA, USA). Routine colony PCR was performed using Taq polymerase (New England Biolabs). All PCR products were cloned into commercially available linearized plasmid pJET1.2/blunt following the manufacturer (Thermo Fischer Scientific, Waltham, MA, USA) and were sequence‐verified using Sanger Sequencing service (Genewiz, CA, USA). Plasmid miniprep kit and gel extraction kit purchased from Qiagen (Hilden, Germany) was used to isolate recombinant plasmids from *E. coli* transformants and to separate PCR products from agarose gels (1% w/v) following the instructions of the manufacturer. Plasmids were electroporated into *P. putida* KT2440 by electroporation, using a 2‐mm cuvette on a Gene Pulser XCell^TM^ (BioRad Labs GmbH, Munich, Germany). Electrocompetent cells for *P. putida* KT2440 were prepared using standard technique, where cells are grown overnight on LB agar plates, followed by resuspension in 300 mM sucrose solution. Cells were washed two times with sucrose solution before making aliquots (100 µl) and their storage at −80°C. Before electroporation, up to 1 µg of the plasmid DNA was added and electroporated using an electroporator with parameters set to voltage 2.5 kV, the capacitance of 25 µF and resistance of 200 Ω. Cells were incubated in LB medium for 2 h for recovery at 30°C with 200 rpm on a shaker incubator before being plated on LB plates with the respective antibiotic(s). Primer designing (SnapGene, GSL Biotech; available at snapgene.com), vector map generation (SnapGene) and gene analysis (NCBI Blast) were performed using basic bioinformatics tools.

### Construction of a duet‐expression vector (pRGPDuo1) from pBBR1 family

Many autonomously replicating vectors for *P. putida* are derived using the origin of replications pBBR1 and pRO1600. The expression vectors derived using these origins of replication are compatible in *P. putda* KT2440 (Silva‐Rocha *et al*., 2013; Cook *et al*., 2018). Previously, we have shown the construction and functionality of dual‐inducible duet‐expression vector pRGPDuo2 based on cryptic plasmid pRO1600 (Gauttam *et al*., 2020). To expand the existing set of expression vectors for metabolic engineering of *P. putida* KT2440, we designed a compatible vector pRGPDuo1 based on the pBBR1 family (Fig. 1A). To establish the compatibility of pRGPDuo1 and pRGPDuo2 (Gauttam *et al*., 2020), all parameters for vector compatibility, such as two different antibiotic resistance markers (Gent^R^ and Kan^R^) and compatible origins of replications (pBBR1 and pRO1600), have been incorporated carefully during vector design. To individually regulate the expression of two genes, the dual‐inducible characteristic of pRGPDuo2 has been retained in pRGPDuo1 by keeping two multiple cloning sites (MCS1 and MCS2), each controlled either by P*_tac_* (IPTG‐inducible) or by P*_tetR/tetA_* (ATc‐inducible).

The expression plasmid pRGPDuo1 was derived from pRGPDuo2. First, the plasmid pRGPDuo2 was modified to construct an intermediate vector pRGPDuo1 v1 by replacing the DNA sequence coding for replication protein (pRO1600) with the nucleotide sequence coding for gentamicin resistance, using the restriction sites MfeI and BsiWI. Therefore, a gentamicin resistance gene was PCR amplified using plasmid pMQ30 (Shanks *et al*., 2009) as a template and duo1GentR‐fwd/rev as primers. The PCR fragment was ligated into a commercially available cloning vector pJET1.2/blunt to construct sub‐cloning vector pJET‐duo1GentR. The vector pRGPDuo1 v1 was built by ligating MfeI/BsiWI‐digested pRGPDuo2 with MfeI/BsiWI‐digested PCR product (gentamicin‐containing region) from pJET‐duo1GentR. The vector pRGPDuo1 v1 still comprises the origin of replication (ColE1) and antibiotic resistance (Kan^R^), making it non‐compatible with pRGPDuo1. Therefore, in the second round of cloning, the fragment containing nucleotide sequences for kanamycin resistance and ColE1 replicon was replaced with nucleotide sequence coding for broad‐host‐range pBBR1 origin that allows the vector to replicate in *E. coli* as well as in *Pseudomonas*. For this round of cloning, the sequence coding for pBBR1 was PCR amplified using plasmid pGEM00003 as a template and duo1BBR1‐fwd/rev as primers with incorporated restriction sites. The PCR product was ligated into a commercially available pJET1.2/blunt vector to construct pJET‐duo1BBR1, where the product has been sequence‐verified. The expression vector pRGPDuo1 was generated by ligating SacI/BstBI‐digested pRGPDuo1 v1 with SacI/BstBI‐digested PCR product (pBBR1‐containing region) from pJET‐duo1BBR1. Similar to its predecessor pRGPDuo2, the newly constructed pRGPDuo1 comprises of two multiple cloning sites (MCS1 and MCS2) distinctly controlled by two different repressor systems (*lacI/*P*_tac_* and *tetR/*P*_tetA/tetR_*), transcriptional terminators, gentamicin resistance marker, broad‐host‐range origin of replication (pBBR1) to allow replication in *E. coli* and *P. putida* (Fig. 1A). The details regarding construction of pRGPDuo1‐derived vectors are mentioned in the supplementary sheet provided.

### Construction of arabinose‐inducible duet‐expression vectors pRGPDuo3 and pRGPDuo4

For constructing expression plasmids pRGPDuo3 and pRGPDuo4, the previously described duet‐expression vectors pRGPDuo1 and pRGPDuo2 (Gauttam *et al*., 2020) were used as parent vectors. Likewise, their predecessors, pRGPDuo3 and pRGPDuo4, were designed to retain the necessary features to be stably maintained in *P. putida* KT2440 when co‐transformed such as distinct antibiotic markers (Gent^R^ and Kan^R^) and compatible replicons (pBBR1 and pRO1600) (Fig. 1B and C). The DNA fragment containing the nucleotide sequence coding for arabinose operon regulatory protein (AraC), followed by araBAD promoter (P*_bad_*) and T7 stem‐loop structure, was amplified with incorporated restriction sites from pBADTrfp using duopBAD‐fwd/rev primers and cloned into pJET1.2/blunt to create sub‐cloning vector pJET‐duoBAD34, where the amplified fragment has been sequence‐verified. The expression plasmid pRGPDuo3 was constructed by ligating NheI/NdeI‐digested pRGPDuo1 (excluding *tetR*‐P*_tetR/tetA_* region) with NheI/NdeI‐digested PCR product (*araC‐*P*_bad_* containing region) from pJET‐duoBAD34. Similarly, pRGPDuo4 was created by ligating NheI/NdeI‐digested pRGPDuo2 (excluding *tetR*‐P*_tetR/tetA_* region) with NheI/NdeI‐digested PCR product (*araC‐P_bad_* containing region) from pJET‐duoBAD34. The details regarding construction of pRGPDuo3‐ and pRGPDuo4‐derived vectors are mentioned in the supplementary sheet provided.

### Measurement of sfGFP and RFP fluorescence

The adapted cells in minimal media (explained in growth section) were used to prepare 48‐well plates (Sarstedt, Germany) with each well containing 250 µL of cell culture. The RFP fluorescence was measured relative to the optical density (OD_600_) using a spectrofluorophotometer TECAN infinite M200 PRO reader (Mannedorf, Switzerland).

Similarly, GFP fluorescence and cell growth were measured simultaneously using a Synergy plate reader (BioTek Instruments, Inc, Winooski, VT, USA). In all cases, the appropriate amount of inducers (IPTG, ATc and arabinose) were added to induce reporter protein's expression from *t* = 0 h (in 48‐well plates only), if required. Following induction, cells were grown at 30°C with continuous shaking for the next 24 h on a spectrofluorophotometer, and fluorescence relative to OD_600nm_ was recorded at an interval of every 20 min. For sfGFP, an excitation wavelength of 485 nm and an excitation wavelength of 535 nm was used. For RFP, an excitation wavelength of 575 nm and an excitation wavelength of 620 nm were used. The optical density (OD_600nm_) was measured simultaneously for each well to calculate GFP fluorescence intensity/OD_600_ and RFP fluorescence intensity/OD_600_ ratio for fluorescence assays. The strains not expressing GFP or RFP were used as a control and respective uninduced counterpart for determining background fluorescence.

### Deletion of *P. putida* aceEF

A previously established method based on the allelic exchange by homologous recombination was used to create *P. putida* KT2440 deletion mutants (Ouyang *et al*., 2007). The method was used to construct a *P. putida* KT2440 deletion mutant for pyruvate dehydrogenase complex (encoded by *aceEF* operon) that catalyses the conversion of pyruvate to acetyl CoA and carbon dioxide. The gene *aceE* encodes for pyruvate dehydrogenase subunit E1, and *aceF* encodes for dihydrolipoamide acetyltransferase. To construct an *aceEF* deletion mutant of *P. putida*, a suicide plasmid pK18mobsacB (Schäfer *et al*., 1994) was used. To create this deletion, the upper (1033) and lower regions (1033 bp) to *aceEF* were amplified using primers pdhflank‐left‐fwd/rev and pdhflank‐right‐fwd/rev respectively. Both the amplified fragments were assembled and ligated into SalI/BamHI‐digested pK18mobsacB to create pRG1 using commercially available NEBuilder HiFi DNA assembly master mix (New England Biolabs) following the instructions from the manufacturer. The HiFi mixture containing assembled fragments (pRG1) was transformed into *E. coli* DH5α competent cells, and transformants were selected on LB agar plates supplemented with kanamycin antibiotic and grown at 30°C. The suicide plasmid pRG1 was electroporated into *P. putida* KT2440, and the transformants with integrated plasmid into the genome were selected for kanamycin resistance. The transformants were grown on YT agar plates containing sucrose (25%) for counterselection. The isolates obtained on sucrose plates were selected due to integrating the insert and recombining the vector out of the genome. These isolates were simultaneously grown on sucrose plates and the LB plates containing kanamycin. The colonies that showed growth on sucrose plate bud did not grow in the presence of kanamycin were selected to be the one with *aceEF* deletion. The deletion of native *aceEF* operon to create knockout *P. putida* KT2440 strain PP31 was confirmed by colony PCR with product size 1201 bp (rather than 5501 bp) using pdhseq1‐fwd/rev as primers.

### Designing sgRNA for gene repression

It is important for an efficient CRISPRi‐mediated gene repression to carefully select spacer sequence for sgRNA designing. The single guided RNA (sgRNA) sequences for gene targeting were designed following the strategy previously described with some modifications (Tan *et al*., 2018). The sgRNA contained a 20‐nucleotide base‐pairing region sharing homology to the targeted DNA followed by dCas9 handle sequence (designated as sgRNA scaffold sequence) to allow dCas9 binding (Fig. S2A and B). The selected 20‐bp region should be specific and unique for each target to avoid off‐target effects and must be followed by a PAM site depending on the source of catalytically inactive Cas9 (NGG for *S. pyogenes* spdCas9 and NNGTGA or NNGCGA for *S. pasteurianus* dCas9). The combination of restriction sites specific for pRGPdCas9bad (PagI, SbfI, NcoI), pRGPspdCas9bad (BamHI, BglII, BsrGI) and pRGPsgRNA (SalI, SbfI, XhoI) were included in the oligonucleotides to facilitate sgRNA cloning (Fig. S2A and B). The 20‐bp homology sequence specific for each gene targeting (included in sgRNAs) in this study, and their details are mentioned in the supplementary sheet provided (Table S5).

### Genomic integration of dCas9

The *dCas9* gene from *S. pasteurianus* was integrated into *P. putida* KT2440 genome as previously described (Tan *et al*., 2018). The Tn7‐based suicide vector pUC18‐miniTn7T‐Lac‐dCas9 was electroporated and integrated into the genome. The gentamicin selection marker was then removed by electroporating pFLP3 as described previously (Choi and Schweizer, 2006). The genomic integration of *dCas9* gene resulted in *P. putida* KT2440 strain PP34.

### Construction of pRGPsgRNA plasmid

The previously developed sgRNA plasmid pBx‐Spas‐sgRNA‐Kan (Tan *et al*., 2018) that utilizes BbsI sites to facilitate sgRNA cloning was modified to construct pRGPsgRNA (Fig. 4A). To build pRGPsgRNA, a synthetic construct (RGPsgRNA fragment) was designed to include specific restriction sites (SalI and SbfI) to assemble multiple sgRNAs into a single plasmid. The synthetic fragment was cloned into pJET to create the sub‐cloning vector pJET‐sgRNA. The vector pRGPsgRNA was constructed by ligating BglII/HindIII‐digested pBx‐Spas‐sgRNA‐Kan with BglII/HindIII‐digested pJET‐sgRNA.

### Construction of the all‐in‐one dual‐inducible CRISPRi vectors (pRGPdCas9 and pRGPdCas9bad and pRGPspdCas9bad)

The dual‐inducible vectors pRGPDuo2 and pRGPDuo4 were used to construct the all‐in‐one fully inducible vectors pRGPdCas9 (Fig. 4B), pRGPdCas9bad (Fig. 4C) and pRGPspdCas9bad (Fig. 4D). These vectors were designed to create a single‐plasmid‐based CRISPRi system that harbours gene encoding for either the dCas9 homolog of *Streptococcus pasteurianus* (dCas9) or of *Streptococcus pyogenes* (spdCas9) under the control of IPTG‐inducible (P*_lac_* or P*_tac_*) promoter and a multiple cloning site controlled by either P*_tetR/tetA_* (pRGPdCas9) and P*_araC/bad_* (pRGPdCas9bad and pRGPspdCas9bad) repressor system for sgRNA cloning. To construct *S. pasteurianus'* dCas9‐based CRISPRi vectors, the sequence for the dCas9 gene (P*_lac_* controlled) was taken from pUC18‐mini‐Tn7 t‐pLac‐dCas9 using MauBI/BamHI restriction enzymes. The digested fragment (dCas9 containing sequence) was ligated into MauBI/BamHI‐digested pRGPDuo2 to create an intermediate vector pRGPdCas9 v1. The vector pRGPdCas9 v1 was modified to include specific restriction sites (NcoI and SbfI) for sgRNA cloning to control the expression through P*_tetR/tetA_* repressor system. For this purpose, sequence (containing P*_tetR/tetA_* and specific recognition sequences) was amplified from pRGPsgRNA using tetR‐fwd/rev as primers. The amplified sequence is digested using MfeI/SpeI and ligated into MfeI/SpeI‐digested pRGPdCas9 v1 to construct pRGPdCas9. The pRGPdCas9 vector was further modified to construct pRGPdCas9bad, where sgRNA expression is controlled by P*_araC/bad_* repressor system instead of P*_tetR/tetA_* repressor system in pRGPdCas9. To construct pRGPdCas9bad, the sequence (containing P*_araC/bad_* and specific recognition sequences) was amplified from pRGPDuo3 using araC‐bad‐fwd/rev as primers. The amplified sequence is digested using MfeI/SpeI and ligated into MfeI/SpeI‐digested pRGPdCas9 to construct pRGPdCas9bad. The plasmid pRGPDuo4 was used as a parent vector to construct *S. pyogenes'* spdCas9‐based pRGPspdCas9bad. The vector pRGPDuo4 was modified to include additional restriction sites (BamHI and SalI) to construct the intermediate vector pRGPDuo4 v1. For this purpose, a 1000‐bp replaceable fragment was amplified from pRGPdCas9 using spdCas9‐fwd/rev as primers with incorporated restriction sites. The amplified fragment is digested using PstI/KpnI and ligated into PstI/KpnI‐digested pRGPDuo4. To construct pRGPspdCas9bad, the sequence for *S. pyogenes*' spdCas9 gene (P*_tac_* controlled) was taken from pRG_dCas9 (Gauttam *et al*., 2019b) using BglII/XhoI restriction enzymes and ligated into BamHI/SalI‐digested pRGPDuo4 v1. The details regarding sgRNA cloning into CRISPRi vectors (pRGPdCas9, pRGPdCas9bad and pRGPspdCas9bad) are mentioned in supplementary sheet provided.

### Accession number

The complete nucleotide sequence of plasmids pRGPDuo1, pRGPDuo3 and pRGPDuo4 have been submitted to the NCBI GenBank with the accession numbers MT304465, MT304466 and MT304467 respectively.

## Funding Information

This work was part of the Department of Energy (DOE) Joint BioEnergy Institute (http://www.jbei.org) supported by the U.S. DOE, Office of Science, Office of Biological and Environmental Research, through Contract No. DE‐AC02‐05CH11231 between Lawrence Berkeley National Laboratory and the U.S. DOE. The United States Government retains and the publisher, by accepting the article for publication, acknowledges that the United States Government retains a non‐exclusive, paid‐up, irrevocable, worldwide licence to publish or reproduce the published form of this manuscript, or allow others to do so, for United States Government purpose.

## Conflict of interests

None declared.

## Ethical approval and consent to participate

Not applicable.

## Supporting information


**Fig. S1**. Kinetic data for recombinant strains harboring pRGPDuo3 and pRGPDuo4‐derived vectors for OD600 (A), sfGFP (B) and RFP (C). sfGFP and RFP values are not normalized against OD_600_. All the strains were induced with IPTG (1 mM) and arabinose (0.2%). The results are representation of data from at least two independent cultivations.
**Fig. S2**. Design of sgRNA coding sequences. Example of sequence for designed synthetic fragment, to target specific gene using CRISPRi for pRGPdCas9bad (A) and pRGPspdCas9bad (B). The sequence consist of 20 base pair gene targeting sequence followed by sgRNA scaffold sequence for dCas9 binding. The recognition sequences for restriction enzyme are specific for sgRNA cloning into CRISPRi vectors (pRGPdCas9bad and pRGPspdCas9bad). Multiplex gene targeting using these vectors require including of promoter sequence between targeting region and BamHI restriction site for each new sgRNA for concatenation.
**Fig. S3**. Vector map for pRGPdCas9bad (A). Enzyme based sgRNA cloning strategy for CRISPRi vector pRGPdCas9bad (and pRGPdCas9) (B). The sgRNA sequences were flanked with restriction site PagI to the 5’ end, and the NcoI and SbfI were introduced at the 3’ end. The sgRNA fragment can be first cloned into cloning vector such as pJET 1.2 blunt (optional step). For sgRNA cloning into CRISPRi vector, the insert was digested with PagI and SbfI and the CRISPRi vector was digested with NcoI and SbfI. The ligation of these fragments restore the original restriction sites (NcoI and SbfI) that can be further used for second round of sgRNA cloning. Therefore, the strategy allows concatenation of multiple sgRNAs in single vector for multiplex gene targeting. A similar strategy was used for cloning sgRNAs in *S. pyogenes* based CRISPRi vector where BglII/BsrGI digested pRGPspdCas9bad was ligated with BamHI/BsrGI digested sgRNA insert.
**Fig. S4**. Growth analysis of *P. putida* KT2440 and its derivative strains in M9 minimal media. (A) The analysis of *P. putida* growth in the presence of different inducers when pre‐cultures were not induced. (B) Growth comparison of *P. putida* strains carrying vectors pRGPsgRNA (PP35), pRGPdCas9 (PP37) and pRGPdCas9bad (PP39) with no sgRNA insert. (C) Growth comparison of *P. putida* strains expressing *aceE* targeting sgRNA when inducers were not added (UI means uniduced). (D) Growth comparison of *P. putida* strains expressing *aceE*‐targeting sgRNA in the presence and absence of inducers when precultures were not induced. (E) Growth comparison of *P. putida* strains expressing *aceE*‐targeting sgRNA in the presence and absence of inducers when precultures were also induced accordingly. Each graph represent the means and standard deviations of results from duplicate cultures. The results are representation of data from at least two independent cultivations.
**Fig. S5**. Growth analysis of *P. putida* KT2440 and its derivative strains in M9 minimal media with different substrates. Comparison of growth for *P. putida* KT2440 with deletion mutant strain PP31 (deletion of *aceEF* operon) in M9 media with glucose (A) and M9 media supplemented with *p*‐coumaric acid (0.5%) (B). Growth comparison of CRISPRi constructs PP49 (pRGPspdCas9bad) and PP50 (pRGPspdCas9bad‐*aceE1*) in M9 glucose (C) and M9 *p*‐coumaric acid (D) when pre‐cultures were not induced.
**Fig. S6**. Growth analysis of *P. putida* KT2440 and its derivative strains in M9 minimal media. Leakiness of *S. pyogenes* spdCas9‐based CRISPRi system (pRGPspdCas9bad) and its effect on growth when pre‐cultures were not induced. Following recombinant strains were analyzed: PP49 (A), PP52 (B), PP56 (C), PP60 (D), PP64 (E), PP65 (F), PP66 (G), PP68 (H), and PP73 (I). For strains’ description refer Table 1. The annotation indicates the presence or absence of inducers: uninduced (no inducer added); induced (presence of both inducers: 1mM IPTG and 0.2% w/v arabinose), + IPTG (the only IPTG was added), and + arabinose (the only arabinose was added). Each graph represents the mean values of biological triplicates from at least two individual cultivations, and error bars represent standard deviations.
**Fig. S7**. Comparison of gene targeting in *P. putida* KT2440 using *S. pasteurianus* and *S. pyogenes* based CRISPRi vectors pRGPdCas9bad (A and B) and pRGPspdCas9bad (C and D), respectively. sgRNAs were designed to downregulate the expression of essential genes, namely, *argB* (A and C) and *eda* (B and D) by targeting the non‐template strand. Growth phenotype was assessed for the strains PP41 (carrying pRGPdCas9bad‐*argB*), PP43 (carrying pRGPdCas9bad‐*eda*), PP54 (carrying pRGPspdCas9bad‐*argB1*), PP59 (carrying pRGPspdCas9bad‐*eda1*) and compared to the respective control strain PP39 (carrying pRGPdCas9bad) or PP49 (pRGPspdCas9bad). In each graph strain PP39 or PP49 is represented as a blue triangle. The green circle in each graph represents the respective strain when pre‐cultures are not induced. The red square in each graph represents the respective strain when pre‐cultures are induced. Each graph represents the mean values of biological triplicates from at least two individual cultivations, and error bars represent standard deviations.
**Fig. S8**. The CRISPRi‐mediated tunable repression of essential genes in *P. putida* KT2440. The expression of dCas9 from *S*. *pasteurianus* is under *P_lac_* control (IPTG‐inducible), and the strong pBAD promoter (arabinose‐inducible) controls sgRNA expression. Growth of recombinant *P. putida* strains PP41 (A), PP42 (B), PP44 (C), PP45 (D), PP46 (E), and PP48 (F) was investigated in the presence of different concentrations of arabinose (ranging from 0 to 0.5% w/v). The dCas9 expression was induced by adding IPTG (1mM) in all strains. For the tunable experiment, pre‐cultures were induced accordingly. Time course consumption of glucose was measured in *P. putida* strains KT2440, PP39, PP42, PP43, PP444, and PP46 under conditions when pre‐cultures were not induced (G), and when pre‐cultures were induced with both inducers (1mM IPTG and 0.2% arabinose) (H). Each graph represents the means and standard deviations of results from duplicate cultures. The results are a representation of data from at least two independent cultivations.
**Fig. S9**. The CRISPRi‐mediated tunable repression of essential genes in *P. putida* KT2440. The expression of dCas9 from *S*. *pasteurianus* is under *P_tac_* control (IPTG inducible) while the strong pBAD promoter (arabinose inducible) controls sgRNA expression. Growth of recombinant *P. putida* strains PP41 (carrying pRGPdCas9bad‐*argB*), PP42 (carrying pRGPdCas9bad‐*argH*), PP44 (carrying pRGPdCas9bad‐*edd*), PP45 (carrying pRGPdCas9bad‐*ftsZ*), PP46 (carrying pRGPdCas9bad‐*pheA*), and PP48 (carrying pRGPdCas9bad‐*pyrF*) was investigated in the presence of different concentrations of IPTG (ranging from 0 mM to 2 mM). The sgRNA expression targeting respective essential gene was induced by adding arabinose (0.2% w/v). For tunable experiment precultures were induced accordingly. Each graph represent the means and standard deviations of results from duplicate cultures. The results are representation of data from at least two independent cultivations.
**Table S1**. Plasmids used in this study.
**Table S2**. Oligonucleotides used in this study. Restriction sites are indicated in bold.
**Table S3**. Synthetic fragments used in this study. Restriction sites and sgRNA scaffold sequence are indicated in bold.
**Table S4**. Normalized relative fluorescence values for recombinant strains.
**Table S5**. The details of the essential genes for *P. putida* KT2440 (Molina‐Henares et al., 2010; Kuepper et al., 2015) whose expression has been downregulated in this study along with the target sequence and the corresponding PAM sites with their location from transcription start site (TSS).
