# Supplementary material for: Development of dual‐inducible duet‐expression vectors for tunable gene expression control and CRISPR interference‐based gene repression in Pseudomonas putida KT2440
Source: Microb Biotechnol. 2021 May 19;14(6):2659–78. doi: 10.1111/1751-7915.13832 (PMC8601191; doi:10.1111/1751-7915.13832)
Supplement: Supplementary file 1 — Fig. S1. Kinetic data for recombinant strains harboring pRGPDuo3 and pRGPDuo4‐derived vectors for OD600 (A), sfGFP (B) and RFP (C). sfGFP and RFP values are not normalized against OD600. All the strains were induced with IPTG (1 mM) and arabinose (0.2%). The results are representation of data from at least two independent cultivations. Fig. S2. Design of sgRNA coding sequences. Example of sequence for designed synthetic fragment, to target specific gene using CRISPRi for pRGPdCas9bad (A) and pRGPspdCas9bad (B). The sequence consist of 20 base pair gene targeting sequence followed by sgRNA scaffold sequence for dCas9 binding. The recognition sequences for restriction enzyme are specific for sgRNA cloning into CRISPRi vectors (pRGPdCas9bad and pRGPspdCas9bad). Multiplex gene targeting using these vectors require including of promoter sequence between targeting region and BamHI restriction site for each new sgRNA for concatenation. Fig. S3. Vector map for pRGPdCas9bad (A). Enzyme based sgRNA cloning strategy for CRISPRi vector pRGPdCas9bad (and pRGPdCas9) (B). The sgRNA sequences were flanked with restriction site PagI to the 5’ end, and the NcoI and SbfI were introduced at the 3’ end. The sgRNA fragment can be first cloned into cloning vector such as pJET 1.2 blunt (optional step). For sgRNA cloning into CRISPRi vector, the insert was digested with PagI and SbfI and the CRISPRi vector was digested with NcoI and SbfI. The ligation of these fragments restore the original restriction sites (NcoI and SbfI) that can be further used for second round of sgRNA cloning. Therefore, the strategy allows concatenation of multiple sgRNAs in single vector for multiplex gene targeting. A similar strategy was used for cloning sgRNAs in S. pyogenes based CRISPRi vector where BglII/BsrGI digested pRGPspdCas9bad was ligated with BamHI/BsrGI digested sgRNA insert. Fig. S4. Growth analysis of P. putida KT2440 and its derivative strains in M9 minimal media. (A) The analysis of P. putida gro [file MBT2-14-2659-s001.docx]

**Development of dual-inducible duet-expression vectors for tunable gene expression control and CRISPR interference-based gene repression in *Pseudomonas putida* KT2440**

Rahul Gauttam^1,2^, Aindrila Mukhopadhyay^1,2^, Blake A. Simmons ^1,2^, Steven W. Singer^1,2^

^1^*The Joint BioEnergy Institute, Emeryville, CA, USA*

*^2^Biological Systems and Engineering Division, Lawrence Berkeley National Laboratory, Berkeley, CA, USA*

* Corresponding author. Mailing address: Lawrence Berkeley National Laboratory, 1 Cyclotron Road, Berkeley, CA, USA, 94720. Phone: +1 510-486-5556. E-mail: swsinger@lbl.gov

**Supplementary Methods**

**Construction of pRGPDuo1-derived vectors:** The gene encoding for sfGFP was amplified using pGEM00003 as a template and duosfGFP-fwd/rev as primers. The nucleotide sequence for RFP was amplified using pBADTrfp as a template and duoRFP-fwd/rev (or badRFP-fwd/rev) as primers. The plasmids pRGPDuo1-sfGFP_tet_ and pRGPDuo1-RFP_tet_ were constructed by ligating NheI/EcoRI-digested pRGPDuo1 with NheI/EcoRI-digested PCR product (sfGFP or RFP containing region). The plasmids pRGPDuo1-sfGFP_tac_ and pRGPDuo1-RFP_tac_ were generated by ligating PstI/BamHI-digested pRGPDuo1 with PstI/BamHI-digested PCR product (sfGFP or RFP containing region).

**Construction of pRGPDuo3 and pRGPDuo4-derived vectors:** To show the functionality of expression vectors pRGPDuo3 and pRGPDuo4, following recombinant plasmids were created expressing reporter proteins (sfGFP and RFP) in different combinations namely, pRGPDuo3-sfGFP_bad_, pRGPDuo3-sfGFP_tac_, pRGPDuo4-sfGFP_tac_, pRGPDuo4-RFP_bad_, pRGPDuo4-RFP_tac_ and pRGPDuo4-sfGFP_bad_. The plasmid pRGPDuo3-sfGFP_tac_ (and pRGPDuo4-sfGFP_tac_) was constructed by ligating PstI/BamHI-digested pRGPDuo3 (and pRGPDuo4) with PstI/BamHI-digested PCR product from pGEM00003 (duosfGFP-fwd/rev). The plasmids pRGPDuo4-RFP_bad_ and pRGPDuo4-sfGFP_bad_ were created by ligating BglII/NheI-digested pRGPDuo4 with BglII/NheI-digested PCR product (RFP or sfGFP containing region) from pBADTrfp (badRFP-fwd/rev) and pGEM00003 (duosfGFP-fwd/rev), respectively. The plasmid pRGPDuo4-RFP_tac_ was created by ligating PstI/BamHI-digested pRGPDuo4 with PstI/BamHI-digested PCR product (RFP containing region) from pBADTrfp (duoRFP-fwd/rev). The plasmid pRGPDuo3-sfGFP_bad_ was created by ligating EcoRI/NheI-digested pRGPDuo3 with EcoRI/NheI-digested PCR product (sfGFP containing region) from pGEM00003 (duosfGFP-fwd/rev).

**Restriction enzyme based sgRNA cloning in CRISPRi vectors:** All-in-one CRISPRi vectors were used to clone sgRNA sequences using a previously established compatible restriction enzyme-based strategy (Gauttam *et al*., 2019b). The strategy allows the concatenation of multiple sgRNAs into a single plasmid for repressing multiple genes (Fig. S3B). The 20 bp homology sequence targeting a specific gene was included in respective forward oligonucleotide (such as *argB*-sgRNA-fwd,) and a common reverse primer (padCas9 sgRNA-rev) was used to amplify all sgRNA inserts. To clone sgRNAs into pRGPdCas9bad, the sgRNA sequences were PCR amplified using pRGPdCas9bad-*aceE* as template and restriction sites (PagI and SbfI) were incorporated in fwd/rev primer pair. To construct these vectors, sgRNA(s) was amplified, and PagI/SbfI-digested insert was ligated into NcoI/SbfI-digested pRGPdCas9bad.

Similarly, to clone sgRNAs into pRGPspdCas9bad, the sgRNA sequences were PCR amplified using PysgRNA template as template and restriction sites (BamHI and BsrGI) were incorporated in fwd/rev primer pair. To construct gene targeting vectors, amplified sgRNAs were BamHI/BsrGI-digested and ligated into BglII/BSrGI-digested pRGPspdCas9bad. To insert sgRNA into pRGPspdCas9bad, the sgRNA sequence was flanked by specific restriction sites (BamHI, BglII, and BsrGI). The BamHI restriction site was placed upstream of the sgRNA sequence, while the recognition sequences for BglII and BsrGI were placed at the 3’-end. The CRISPRi vector (pRGPspdCas9bad) was digested using BglII and BsrGI, and the sgRNA insert was digested using BamHI and BsrGI. The ligation of vector and insert recreates the original restriction sites (BglII and BsrGI) that can be used again for the next round of sgRNA cloning. Similarly, to clone sgRNAs in pRGPspdCas9bad a combination of BglII/BsrGI (for vector digestion) and BamHI/BsrGI (for sgRNA digestion) were used. For more details regarding sgRNA design and sgRNA cloning refer supplementary provided.

**Cloning strategy for *aceE*-targeting CRISPRi vectors:** To clone sgRNAs in dCas9-derived vectors, three synthetic sgRNA fragments (Table S3) were designed with the same 20 bp homologous region to non-template (NT) strand and specific restriction sites depending on the vector to be cloned under the respective inducible promoter system. For instance, XhoI/SbfI-digested synthetic construct *aceE*-sgRNA1 (ATc-inducible) was ligated into SalI/SbfI-digested pRGPsgRNA to create pRGPsgRNA-*aceE*. Similarly, PagI/SbfI digested synthetic constructs *aceE*tetR-sgRNA2 (ATc-inducible) and *aceE*bad-sgRNA3 (arabinose-inducible) were ligated into NcoI/SbfI-digested pRGPdCas9 and pRGPdCas9bad to create pRGPdCas9-*aceE* and pRGPdCas9bad-*aceE* respectively. To target the same gene using spdCas9-derived pRGPspdCas9bad vector, *aceE* specific sgRNA was cloned using PysgRNA template (Table S3) with aceE1 sgRNA-fwd and pydCas9 sgRNA-rev as primers to construct pRGPspdCas9bad-*aceE1*.

**Supplementary Tables**

**Table S1:** Plasmids used in this study.

| **Plasmid(*)** | **Relevant characteristics** | **Source / Reference** |
| --- | --- | --- |
| pGEM00003 | Carrying the gene coding for sfGFP | Gauttam *et al*., 2020 |
| pMQ30 | oriColE1, aac1, ARSH4, CEN6, lacZa, sacB, oriT | Shanks *et al*., 2009 |
| pBADTrfp (JPUB_001229) | pBADrfp derivative, *P_BAD_*_T7 stem-loop_rfp | Bi *et al*., 2013 |
| pJET1.2/blunt | Linearized cloning vector for use in *E. coli*; Amp^R^ | CloneJET PCR Coning Kit (Thermo Scientific) |
| pJET-duo1GentR | pJET1.2/blunt carrying gene for gentamycin resistance; Amp^R^ | This study |
| pJET-duo1BBR1 | pJET1.2/blunt carrying gene for BBR1 (OriV*_P. putida_*); Amp^R^ | This study |
| pJET-duoBAD34 | pJET1.2/blunt carrying DNA sequence for *araC*, pBAD and T7 stem-loop | This study |
| pRGPDuo1 v1 | pRGPDuo2 carrying gene for gentamycin resistance; Gent^R^ | This study |
| pRGPDuo1 | dual-inducible *E. coli/P. putida* shuttle vector (*P_tac_*, *lacI^Q^,* OriV*_P. putida_* (pBBR1), OriV*_E. coli_* (pBBR1), *P_tetR/tetA_, tetR*); Gent^R^ | This study |
| pRGPDuo2 | dual-inducible *E. coli/P. putida* shuttle vector (*P_tac_*, *lacI^Q^,* OriV*_P. putida_* (pRO1600), OriV*_E. coli_* (colE1), *P_tetR/tetA_, tetR*); Kan^R^ | Gauttam *et al*., 2020 |
| pRGPDuo3 | dual-inducible *E. coli/P. putida* shuttle vector (*P_tac_*, *lacI^Q^,* OriV*_P. putida_* (pBBR1), OriV*_E. coli_* (pBBR1), *P_bad_, araC*); Gent^R^ | This study |
| pRGPDuo4 | dual-inducible *E. coli/P. putida* shuttle vector (*P_tac_*, *lacI^Q^,* OriV*_P. putida_* (pRO1600), OriV*_E. coli_* (colE1), *P_bad_, araC*); Kan^R^ | This study |
| pRGPDuo1-sfGFP_tet_ | pRGPDuo1 carrying the gene for super folder green fluorescent protein (sfGFP) under the control of *P_tetR/tetA_*; Gent^R^ | This study |
| pRGPDuo1-sfGFP_tac_ | pRGPDuo1 carrying the gene for super folder green fluorescent protein (sfGFP) under the control of *P_tac_*; Gent^R^ | This study |
| pRGPDuo1-RFP_tac_ | pRGPDuo1 carrying the gene for red fluorescent protein (RFP) under the control of *P_tac_*; Gent^R^ | This study |
| pRGPDuo1-RFP_tet_ | pRGPDuo1 carrying the gene for red fluorescent protein (RFP) under the control of *P_tetR/tetA_*; Gent^R^ | This study |
| pRGPDuo2-RFP_tet_ | pRGPDuo2 carrying the gene for red fluorescent protein (RFP) under the control of *P_tetR/tetA_*; Kan^R^ | Gauttam *et al*., 2020 |
| pRGPDuo3-sfGFP_bad_ | pRGPDuo3 carrying the gene for super folder green fluorescent protein (sfGFP) under the control of *P_bad_*; Gent^R^ | This study |
| pRGPDuo3-sfGFP_tac_ | pRGPDuo3 carrying the gene for super folder green fluorescent protein (sfGFP) under the control of *P_tac_*; Gent^R^ | This study |
| pRGPDuo4-sfGFP_bad_ | pRGPDuo4 carrying the gene for super folder green fluorescent protein (sfGFP) under the control of *P_bad_*; Kan^R^ | This study |
| pRGPDuo4-sfGFP_tac_ | pRGPDuo4 carrying the gene for super folder green fluorescent protein (sfGFP) under the control of *P_tac_*; Kan^R^ | This study |
| pRGPDuo4-RFP_bad_ | pRGPDuo4 carrying the gene for red fluorescent protein (RFP) under the control of *P_bad_*; Kan^R^ | This study |
| pRGPDuo4-RFP_tac_ | pRGPDuo4 carrying the gene for red fluorescent protein (RFP) under the control of *P_tac_*; Kan^R^ | This study |
| pK18mobSacB (JPUB_011084) | Suicide plasmid for gene knockout; Kan^R^ | Schafer *et al*., 1994 |
| pRG1 | For gene *aceF* – *aceE* (PP0338 – PP0339) knockout; Kan^R^ | This study |
| pUC18-mini-Tn7t-pLac-dCas9 | Tn7 integrating plasmid containing gene *S. pasteurianus* dCas9 expressed from the Plac promoter for expression in *P. putida* (Addgene no. 105235) | Tan *et al*., 2018 |
| pFLP3 | Flp mediated excision marker | Tan *et al*., 2018 |
| pBx-Spas-sgRNA-Kan | Vector for sgRNA cloning (Addgene no. 105233); Kan^R^ | Tan *et al*., 2018 |
| pJET-sgRNA | pJET1.2/blunt carrying synthetic fragment RGPsgRNA | This study |
| pRGPsgRNA | A modified pBx-Spas-sgRNA-Kan plasmid to express the coding sequence for targeting sgRNA and the expression was controlled by ATc inducible tetR/*P_tetR/tetA_* repressor system; Kan^R^ | This study |
| pJET-*aceE*sgRNA1 | pJET1.2/blunt carrying synthetic fragment *aceE*sgRNA1 | This study |
| pRGPsgRNA-*aceE* | pRGPsgRNA carrying the sgRNA coding sequence targeting *aceE*; Kan^R^ | This study |
| pRGPdCas9 v1 | pRGPDuo2 carrying gene encoding dCas9 protein from *S. pasteurianus*; Kan^R^ | This study |
| pJET-*aceE*sgRNA2 | pJET1.2/blunt carrying synthetic fragment *aceE*sgRNA2 | This study |
| pRGPdCas9 | dual-inducible CRISPRi system carrying two repressor genes (*lacI, tetR*) and gene encoding the dCas9 protein from *S. pasteurianus* under the control of IPTG-inducible P*_lac_* promoter and a cloning site for sgRNA cloning under the control of Atc-inducible P*_tet_* promoter; Kan^R^ | This study |
| pJET-*aceE*sgRNA3 | pJET1.2/blunt carrying synthetic fragment *aceE*sgRNA3 | This study |
| pRGPdCas9bad | dual-inducible CRISPRi system carrying two repressor genes (*lacI, araC*) and gene encoding the dCas9 protein from *S. pasteurianus* under the control of IPTG-inducible P*_lac_* promoter and a cloning site for sgRNA cloning under the control of arabinose-inducible P*_aracbad_* promoter; Kan^R^ | This study |
| pRGPdCas9-*aceE* | pRGPdCas9 carrying the sgRNA coding sequence targeting *aceE*; Kan^R^ | This study |
| pRGPdCas9bad-*argB* | pRGPdCas9bad carrying the sgRNA coding sequence targeting *argB*; Kan^R^ | This study |
| pRGPdCas9bad-*argH* | pRGPdCas9bad carrying the sgRNA coding sequence targeting *argH*; Kan^R^ | This study |
| pRGPdCas9bad-*eda* | pRGPdCas9bad carrying the sgRNA coding sequence targeting *eda*; Kan^R^ | This study |
| pRGPdCas9bad-*edd* | pRGPdCas9bad carrying the sgRNA coding sequence targeting *edd*; Kan^R^ | This study |
| pRGPdCas9bad-*ftsZ* | pRGPdCas9bad carrying the sgRNA coding sequence targeting *ftsZ*; Kan^R^ | This study |
| pRGPdCas9bad-*pheA* | pRGPdCas9bad carrying the sgRNA coding sequence targeting *pheA*; Kan^R^ | This study |
| pRGPdCas9bad-*pyrF* | pRGPdCas9bad carrying the sgRNA coding sequence targeting *pyrF*; Kan^R^ | This study |
| pRGPdCas9bad-*trpG* | pRGPdCas9bad carrying the sgRNA coding sequence targeting *trpG*; Kan^R^ | This study |
| pRG_dCas9 | *S. pyogenes* dCas9 based CRISPRi vector | Gauttam *et al.,* 2019 |
| pRGPDuo4 v1 | pRGPDuo4 with additional restriction sites BamHI and SalI in MCS1 | This study |
| pRGPspdCas9bad | dual-inducible CRISPRi system carrying two repressor genes (*lacI, araC*) and gene encoding the dCas9 protein from *Streptococcus pyogenes* under the control of IPTG-inducible P*_tac_* promoter and a cloning site for sgRNA cloning under the control of arabinose-inducible P*_araCbad_* promoter; Kan^R^ | This study |
| pRGPspdCas9bad-*aceE1* | pRGPspdCas9bad carrying the sgRNA1 targeting *aceE*; Kan^R^ | This study |
| pRGPspdCas9bad-*aceE2* | pRGPspdCas9bad carrying the sgRNA2 targeting *aceE*; Kan^R^ | This study |
| pRGPspdCas9bad-*aceE3* | pRGPspdCas9bad carrying the sgRNA3 targeting *aceE*; Kan^R^ | This study |
| pRGPspdCas9bad-*aceE4* | pRGPspdCas9bad carrying the sgRNA4 targeting *aceE*; Kan^R^ | This study |
| pRGPspdCas9bad-*argB1* | pRGPspdCas9bad carrying the sgRNA1 targeting *argB*; Kan^R^ | This study |
| pRGPspdCas9bad-*argH1* | pRGPspdCas9bad carrying the sgRNA1 targeting *argH*; Kan^R^ | This study |
| pRGPspdCas9bad-*argH2* | pRGPspdCas9bad carrying the sgRNA2 targeting *argH*; Kan^R^ | This study |
| pRGPspdCas9bad-*argH3* | pRGPspdCas9bad carrying the sgRNA3 targeting *argH*; Kan^R^ | This study |
| pRGPspdCas9bad-*argH4* | pRGPspdCas9bad carrying the sgRNA4 targeting *argH*; Kan^R^ | This study |
| pRGPspdCas9bad-*eda1* | pRGPspdCas9bad carrying the sgRNA1 targeting *eda*; Kan^R^ | This study |
| pRGPspdCas9bad-*edd1* | pRGPspdCas9bad carrying the sgRNA1 targeting *edd*; Kan^R^ | This study |
| pRGPspdCas9bad-*edd2* | pRGPspdCas9bad carrying the sgRNA2 targeting *edd*; Kan^R^ | This study |
| pRGPspdCas9bad-*edd3* | pRGPspdCas9bad carrying the sgRNA3 targeting *edd*; Kan^R^ | This study |
| pRGPspdCas9bad-*edd4* | pRGPspdCas9bad carrying the sgRNA4 targeting *edd*; Kan^R^ | This study |
| pRGPydCas9bad-*ftsZ1* | pRGPspdCas9bad carrying the sgRNA1 targeting *ftsZ*; Kan^R^ | This study |
| pRGPspdCas9bad-*pheA1* | pRGPspdCas9bad carrying the sgRNA1 targeting *pheA*; Kan^R^ | This study |
| pRGPspdCas9bad-*pheA2* | pRGPspdCas9bad carrying the sgRNA2 targeting *pheA*; Kan^R^ | This study |
| pRGPspdCas9bad-*pheA3* | pRGPspdCas9bad carrying the sgRNA3 targeting *pheA*; Kan^R^ | This study |
| pRGPspdCas9bad-*pheA4* | pRGPspdCas9bad carrying the sgRNA4 targeting *pheA*; Kan^R^ | This study |
| pRGPspdCas9bad-*pyrF1* | pRGPspdCas9bad carrying the sgRNA1 targeting *pyrF*; Kan^R^ | This study |
| pRGPspdCas9bad-*pyrF2* | pRGPspdCas9bad carrying the sgRNA2 targeting *pyrF*; Kan^R^ | This study |
| pRGPspdCas9bad-*pyrF3* | pRGPspdCas9bad carrying the sgRNA3 targeting *pyrF*; Kan^R^ | This study |
| pRGPspdCas9bad-*pyrF4* | pRGPspdCas9bad carrying the sgRNA4 targeting *pyrF*; Kan^R^ | This study |
| pRGPspdCas9bad-*trpG1* | pRGPspdCas9bad carrying the sgRNA1 targeting *trpG*; Kan^R^ | This study |
| pRGPspdCas9bad-*trpG2* | pRGPspdCas9bad carrying the sgRNA2 targeting *trpG*; Kan^R^ | This study |
| pRGPspdCas9bad-*trpG3* | pRGPspdCas9bad carrying the sgRNA3 targeting *trpG*; Kan^R^ | This study |
| pRGPspdCas9bad-*trpG4* | pRGPspdCas9bad carrying the sgRNA4 targeting *trpG*; Kan^R^ | This study |

**Table S2:** Oligonucleotides used in this study. Restriction sites are indicated in bold.

| **Oligonucleotides** | **Sequence (5’ → 3’)** | **Plasmid construction / purpose** |
| --- | --- | --- |
| duo1GentR-fwd | tcacg**caattg**cttgacataagcctgttcggttcg | Construction of pRGPDuo1 v1 using pMQ30 and pRGPDuo2 |
| duo1GentR-rev | acgtg**cgtacg**cccttaggtggcggtacttggg |  |
| duo1BBR1-fwd | agcatggtaccac**gagctc**ccaaactcgagtaaggatctccag | Construction of pRGPDuo1 using pRGPDuo1 v1 and pGEM00003 |
| duo1BBr1-rev | atttcgaaagc**ttcgaa**cgcttggactcctgttgatagatcca |  |
| duosfGFP-fwd | ctgcagag**ctgcaggaattcagatct**gattaaagaggagaaattaagcatgagcaaagg | Construction of pRGPDuo3-sfGFP_tac_, pRGPDuo3-sfGFP_bad_, pRGPDuo4-sfGFP_tac_, and pRGPDuo4-sfGFP_bad_ |
| duosfGFP-rev | gctagcagct**gctagc**ttt**ggatcc**ttatttgtagagctcatcca |  |
| duoRFP-fwd | aacgc**ctgcag**gcttttaagaaggagatatacatatggcgagtagcg | Construction of pRGPDuo1-RFP_tet_, pRGPDuo1-RFP_tac_ and pRGPDuo4-RFP_tac_ |
| duoRFP-rev | ggagatccttactcgagttt**ggatcc**t |  |
| duopBAD-fwd | catatggaattc**catatg**tgcagggcttcccaaccttacc | Construction of pRGPDuo3 and pRGPDuo4 |
| duopBAD-rev | agc**gctagc**gcggccgcaccatggagatcttttgaattccaaaattatttctagagggaa |  |
| badRFP-fwd | gaattc**gaattc**acg**agatct**ag**ctgcag**gcttttaagaaggagatatacatatggcgag | Construction of pRGPDuo3-RFP_bad_ and pRGPDuo4-RFP_bad_ |
| badRFP-rev | gctagcacg**gctagc**ct**ggatcc**taagcaccggtggagtgac |  |
| pdhflank-left-fwd | gccaagcttgcatgcctgcaggccttcggtttcaccgccc |  |
| pdhflank-left-rev | agtgtgctggcggcttgctccagggcgga | To create the knockout *P. putida* KT2440 strain (PP31) for *ΔaceEF* |
| pdhflank-right-fwd | tggagcaagccgccagcacactgcccctc |  |
| pdhflank-right-rev | gaattcgagctcggtacccggggcggtaccaggcgatgatc |  |
| pdhseq1-fwd | cctgcaggtcgagttcggg | To confirm *ΔaceEF* deletion in PP31 |
| pdhseq1-rev | ctgctggtcatcgagcacg |  |
| tetR-fwd | agctat**caattg**accatggcgaaatgaccgaccaagcgac | To construct pRGPdCas9 using pRGPsgRNA and pRGPdCas9 v1 |
| tetR-rev | agcataactagtggcgaattggagctccaccg |  |
| araC-bad-fwd | aag**actagt**aaactagtttgcagggcttcccaacctt | To construct pRGPdCas9bad using pRGPDuo3 and pRGPdCas9 |
| araC-bad-rev | tag**gaattc**acgcctgcaggacgaccatggaccaaaattatttctagagggaaaccgttg |  |
| **Oligonucleotides used for sgRNA cloning into *S. pasteurianus* dCas9 based CRISPRi vector (pRGPdCas9bad)** | | |
| padCas9 sgRNA-rev | atgcataata**atgcat**taaggacactgtatctgcgtcccac | Common reverse primer for sgRNA cloning in pRGPdCas9bad |
| argB sgRNA-fwd | TCAT**TCATGA**CGGCTACATGGGAAGCGGCAgtttttgtactcgaaagagcctacaaaga | pRGPdCas9bad-*argB* |
| argH sgRNA-fwd | TCAT**TCATGA**ATCTACCGAGGCGGTGAAACgtttttgtactcgaaagagcctacaaaga | pRGPdCas9bad-*argH* |
| eda sgRNA-fwd | AT**TCATGA**GGCACCAGTAGATGTCCAGGGGGgtttttgtactcgaaagagcctacaaaga | pRGPdCas9bad-*eda* |
| edd sgRNA-fwd | TCA**TCATGA**GCTGCGTTCGACCAGCCGCTgtttttgtactcgaaagagcctacaaaga | pRGPdCas9bad-*edd* |
| ftsZ sgRNA-fwd | TCA**TCATGA**CTTCGATGCTGCTCTTGACCgtttttgtactcgaaagagcctacaaaga | pRGPdCas9bad-*ftsZ* |
| pheA sgRNA-fwd | TCAT**TCATGA**ACCGGCTTCTCGCCTTCTTTgtttttgtactcgaaagagcctacaaaga | pRGPdCas9bad-*pheA* |
| pyrF sgRNA-fwd | TCAT**TCATGA**CTCACGGGTAGGGAAATCCAgtttttgtactcgaaagagcctacaaaga | pRGPdCas9bad-*pyrF* |
| trpG sgRNA-fwd | TCATG**TCATGA**AAGGTACTGAACAACGTTGTgtttttgtactcgaaagagcctacaaaga | pRGPdCas9bad-*trpG* |
| **Oligonucleotides used for sgRNA cloning into *S. pyogenes* dCas9 based CRISPRi vector (pRGPspdCas9bad)** | | |
| spdCas9-fwd | gcc**ctgcag**tgcatgcctgcaggatccag**ggatcc**GAAGGGCCTGGGCAAGAAGAAG | Construction of pRGPDuo4spdCas9 v1 |
| spdCas9-rev | agtg**ggtacc**gagctcggtacgtcgacct**gtcgac**GTCCGAGTACTTCAGGCCCATC |  |
| pydCas9 sgRNA-rev | taca**tgtaca**acgta**agatct**cggaaacaacaaaaggatgtcgactat | Common reverse primer for sgRNA cloning in pRGPspdCas9bad |
| aceE1 sgRNA-fwd | ccta**ggatcc**CCTGGGTTTCGATTGGATCGgttttagagctagaaatagcaagttaaaat | pRGPspdCas9bad-*aceE1* |
| aceE2 sgRNA-fwd | ccta**ggatcc**GTCTTGCATGGCTTGCTCCAgttttagagctagaaatagcaagttaaaat | pRGPspdCas9bad-*aceE2* |
| aceE3 sgRNA-fwd | ccta**ggatcc**CAGGGCATCCAGCCATTCCTgttttagagctagaaatagcaagttaaaat | pRGPspdCas9bad-*aceE3* |
| aceE4 sgRNA-fwd | ccta**ggatcc**GCCAGGCATGCGTGCTTCGTgttttagagctagaaatagcaagttaaaat | pRGPspdCas9bad-*aceE4* |
| argB1 sgRNA-fwd | ccta**ggatcc**GGGAAGCGGCATCGCGATCGgttttagagctagaaatagcaagttaaaat | pRGPspdCas9bad-*argB1* |
| argH1 sgRNA-fwd | ccta**ggatcc**CCAGGACTGATTGGTCTTCTgttttagagctagaaatagcaagttaaaat | pRGPspdCas9bad-*argH1* |
| argH2 sgRNA-fwd | ccta**ggatcc**CTCATGGATTCACTCATTGCgttttagagctagaaatagcaagttaaaat | pRGPspdCas9bad-*argH2* |
| argH3 sgRNA-fwd | ccta**ggatcc**CGGGCGACGAAGGCGTCGACgttttagagctagaaatagcaagttaaaat | pRGPspdCas9bad-*argH3* |
| argH4 sgRNA-fwd | ccta**ggatcc**TGCCGGCTTCAATCTCGCCCgttttagagctagaaatagcaagttaaaat | pRGPspdCas9bad-*argH4* |
| eda1 sgRNA-fwd | ccta**ggatcc**TTCAAGGGTGGTCATGACTGgttttagagctagaaatagcaagttaaaat | pRGPspdCas9bad-*eda1* |
| edd1 sgRNA-fwd | ccta**ggatcc**TGGGTGACCTCAAGGATGCGgttttagagctagaaatagcaagttaaaata | pRGPspdCas9bad-*edd1* |
| edd2 sgRNA-fwd | ccta**ggatcc**GGATGCATGTACTGGACTCCgttttagagctagaaatagcaagttaaaat | pRGPspdCas9bad-*edd2* |
| edd3 sgRNA-fwd | ccta**ggatcc**GCTGCGTTCGACCAGCCGCTgttttagagctagaaatagcaagttaaaat | pRGPspdCas9bad-*edd3* |
| edd4 sgRNA-fwd | ccta**ggatcc**GAAGTGCAAGTAAGGCTGGTgttttagagctagaaatagcaagttaaaat | pRGPspdCas9bad-*edd4* |
| ftsZ1 sgRNA-fwd | ccta**ggatcc**ACTTTAATGACCGGACTTTGgttttagagctagaaatagcaagttaaaat | pRGPspdCas9bad-*ftsZ1* |
| pheA1 sgRNA-fwd | ccta**ggatcc**CAGCGCCTTGAGCTCCTGTTgttttagagctagaaatagcaagttaaaat | pRGPspdCas9bad-*pheA1* |
| pheA2 sgRNA-fwd | ccta**ggatcc**CCAGCGCTTCTACCGCTTCCgttttagagctagaaatagcaagttaaaat | pRGPspdCas9bad-*pheA2* |
| pheA3 sgRNA-fwd | ccta**ggatcc**GCTCGAGAATTTTCTCGTCGgttttagagctagaaatagcaagttaaaat | pRGPspdCas9bad-*pheA3* |
| pheA4 sgRNA-fwd | ccta**ggatcc**CCATCTCTTCGTTGCCCAGCgttttagagctagaaatagcaagttaaaat | pRGPspdCas9bad-*pheA4* |
| pyrF1 sgRNA-fwd | ccta**ggatcc**GGGAAATCCAGGGCGACGATCgttttagagctagaaatagcaagttaaaat | pRGPspdCas9bad-*pyrF1* |
| pyrF2 sgRNA-fwd | ccta**ggatcc**CTGGCAGGCGGACATGGGCAgttttagagctagaaatagcaagttaaaat | pRGPspdCas9bad-*pyrF2* |
| pyrF3 sgRNA-fwd | ccta**ggatcc**GATCAGGGGCGTCTGGCAGGgttttagagctagaaatagcaagttaaaat | pRGPspdCas9bad-*pyrF3* |
| pyrF4 sgRNA-fwd | ccta**ggatcc**GACCATCCACACGCCCATCTgttttagagctagaaatagcaagttaaaat | pRGPspdCas9bad-*pyrF4* |
| trpG1 sgRNA-fwd | ccta**ggatcc**ATTGTCGATCATCAGTAACAgttttagagctagaaatagcaagttaaaat | pRGPspdCas9bad-*trpG1* |
| trpG3 sgRNA-fwd | ccta**ggatcc**ACTTCATGATTCGAACCTTCgttttagagctagaaatagcaagttaaaat | pRGPspdCas9bad-*trpG2* |
| trpG3 sgRNA-fwd | ccta**ggatcc**CCTCGGCACCCAGCTCGCCAgttttagagctagaaatagcaagttaaaat | pRGPspdCas9bad-*trpG3* |
| trpG4 sgRNA-fwd | ccta**ggatcc**GCCCGATTGACTGGTGGCCCgttttagagctagaaatagcaagttaaaat | pRGPspdCas9bad-*trpG4* |

**Table S3:** Synthetic fragments used in this study. Restriction sites and sgRNA scaffold sequence are indicated in bold.

| **Synthetic fragment** | **Sequence (5’ → 3’)** | **Purpose** |
| --- | --- | --- |
| RGPsgRNA fragment | ggggatca**agatct**gatcaagagacaggatgaggatcgtttcgcatgattgaacaagatggattgcacgcaggttctccggccgcttgggtggagaggctattcggctatgactgggcacaacagacaatcggctgctctgatgccgccgtgttccggctgtcagcgcaggggcgcccggttctttttgtcaagaccgacctgtccggtgccctgaatgaactgcaggacgaggcagcgcggctatcgtggctggccacgacgggcgttccttgcgcagctgtgctcgacgttgtcactgaagcgggaagggactggctgctattgggcgaagtgccggggcaggatctcctgtcatctcaccttgctcctgccgagaaagtatccatcatggctgatgcaatgcggcggctgcatacgcttgatccggctacctgcccattcgaccaccaagcgaaacatcgcatcgagcgagcacgtactcggatggaagccggtcttgtcgatcaggatgatctggacgaagagcatcaggggctcgcgccagccgaactgttcgccaggctcaaggcgcgcatgcccgacggcgaggatctcgtcgtgacccatggcgatgcctgcttgccgaatatcatggtggaaaatggccgcttttctggattcatcgactgtggccggctgggtgtggcggaccgctatcaggacatagcgttggctacccgtgatattgctgaagagcttggcggcgaatgggctgaccgcttcctcgtgctttacggtatcgccgctcccgattcgcagcgcatcgccttctatcgccttcttgacgagttcttctgagcgggactctggggttcgaaatgaccgaccaagcgacgcccaacctgccatcacgagatttcgattccaccgccgccttctatgaaaggttgggcttcggaatcgtttttccgggacatcgtcgacgccatcgcctgcagggcatctgcggccgcgaggtacctgatccggatttgataaaacgaaaggcccagtctttcgactgagcctttcgttttataaacgttatccaaGGCTCACCTTCGGGTGGGCCTTTCTGCGcatgtcggcagaatgcttaatgaattacaacagtttttatat**aagctt**taa | Construction of pRGPsgRNA |
| *aceE*-sgRNA1 | a**ctcgag**gggatctcatgctggagttcttcgcccacccccatgggcaaattgacatccctatcagtgatagagatactgagcacacgtcttcatgcagctgaagacttGATGGTGTTGCGGTATGGCG**gtttttgtactcgaaagagcctacaaagataaggctttatgccgaattcaagcaccccatgttttgacatgaggtgctttttttt**aa**gtcgac**acc**cctgcagg**cg | Construction of pRGPsgRNA-*aceE* |
| *aceEtetR-sgRNA2* | a**tcatga**aattgacatccctatcagtgatagagatactgagcacacgtcttcatgcagctgaagacttGATGGTGTTGCGGTATGGCG**gtttttgtactcgaaagagcctacaaagataaggctttatgccgaattcaagcaccccatgttttgacatgaggtgctttttttt**aagtcacac**ccatgg**agct**cctgcagg**catg | Construction of pRGPdCas9-*aceE* |
| *aceEbad-sgRNA3* | a**tcatga**aattgacatactgagcacacgtcttcatgcagctgaagacttGATGGTGTTGCGGTATGGCG**gtttttgtactcgaaagagcctacaaagataaggctttatgccgaattcaagcaccccatgttttgacatgaggtgctttttttt**aagtcacac**ccatgg**agct**cctgcagg**catg | Construction of pRGPdCas9bad-*aceE* |
| *PysgRNA template* | gatccCCTGGGTTTCGATTGGATCG**gttttagagctagaaatagcaagttaaaataaggctagtccgttatcaacttgaaaaagtggcaccgagtcggtgctttttttatttttt**gtcactattgttatgtaaaatgccacctctgacagtatggaacgcaaacttctgtctagtggatagtcgacatccttttgttgtttccgggtgtacaatatggacttcctcttttctggcaaccaaacccatacatcgggattcctataataccttcgttggtctccctaacatgtaggtggcggaggggagatatacaatagaacagataccagacaagacataatgggctaaacaagactacaccaattacactgcctcattgatggtggtacataacgaactaatactgtagccctagacttgatagccatcatcatatcgaagtttcactaccctttttccatttgccatctattgaagtaataataggcgcatgcaacttcttttctttttttttcttttctctctcccccgttgttgtctcaccatatccgcaatgacaaaaaaatgatggaagacactaaaggaaaaaattaacgacaaagacagcaccaacagatgtcgttgttccagagct | Sequence template for sgRNA amplification for cloning into pRGPspdCas9bad |

**Table S4:**

| **Strain** | **GFP/OD_600_** | **RFP/OD_600_** |  | **Strain** | **GFP/OD_600_** | **RFP/OD_600_** |  | **Strain** | **GFP/OD_600_** | **RFP/OD_600_** |
| --- | --- | --- | --- | --- | --- | --- | --- | --- | --- | --- |
| PP7 UI | 410 ± 9 | 683 ± 15 |  | PP7 Ind | 370 ± 15 | 705 ± 14 |  | PP19 UI | 416 ± 21 | 688 ± 38 |
| PP8 UI | 818 ± 60 | 730 ± 23 |  | PP8 Ind | 2435 ± 58 | 770 ± 15 |  | PP19 Ind | 392 ± 6 | 4281 ± 56 |
| PP9 UI | 1258 ± 64 | 696 ± 10 |  | PP9 Ind | 2059 ± 98 | 590 ± 12 |  | PP20 Ind | 335 ± 15 | 679 ± 15 |
| PP10 UI | 537 ± 35 | 1253 ± 74 |  | PP10 Ind | 474 ± 28 | 5341 ± 419 |  | PP21 Ind | 180 ± 15 | 401 ± 8 |
| PP11 UI | 308 ± 5 | 687 ± 24 |  | PP11 Ind | 333 ± 32 | 4207 ± 9 |  | PP22 Ind | 219 ± 21 | 409± 13 |
| PP12 UI | 420 ± 9 | 682 ± 6 |  | PP12 Ind | 488 ± 12 | 739 ± 13 |  | PP23 Ind | 391 ± 20 | 410 ± 13 |
| PP13 UI | 398 ± 13 | 819 ± 18 |  | PP13 Ind | 346 ± 7 | 662 ± 44 |  | PP24 Ind | 2369 ± 301 | 3483 ± 431 |
| PP14 UI | 1402 ± 114 | 733 ± 22 |  | PP14 Ind | 5923 ± 230 | 734 ± 32 |  | PP25 Ind | 2441 ± 353 | 4683 ± 231 |
| PP15 UI | 1800 ± 132 | 594 ± 16 |  | PP15 Ind | 4956 ± 380 | 669 ± 38 |  | PP26 Ind | 2512 ± 463 | 4683 ± 233 |
| PP16 UI | 458 ± 20 | 678 ± 43 |  | PP16 Ind | 4257 ± 143 | 748 ± 9 |  | PP27 Ind | 3011 ± 489 | 5675 ± 413 |
| PP17 UI | 841 ± 50 | 840 ± 43 |  | PP17 Ind | 2692 ± 119 | 851 ± 9 |  | PP28 Ind | 2176 ± 376 | 5357 ± 410 |
| PP18 UI | 483 ± 18 | 835 ± 9 |  | PP18 Ind | 526 ± 14 | 9415 ± 733 |  |  |  |  |

**UI:** No Inducer added; **Ind:** Inducer added

**Table S5:** The details of the essential genes for *P. putida* KT2440 ((Molina-Henares *et al*., 2010; Kuepper *et al*., 2015)) whose expression has been downregulated in this study along with the target sequence and the corresponding PAM sites with their location from transcription start site (TSS).

| **Target gene** | **PAM site** | **Strains** | **PAM location from TSS** | **Target sequence** |
| --- | --- | --- | --- | --- |
| pyruvate dehydrogenase E1 component (*aceE*) | GTGA | PP40 | + 148 | GATGGTGTTGCGGTATGGCG |
|  | AGG | PP50 | + 8 | CCTGGGTTTCGATTGGATCG |
|  | GGG | PP51 | -11 | GTCTTGCATGGCTTGCTCCA |
|  | GGG | PP52 | + 25 | CAGGGCATCCAGCCATTCCT |
|  | GGG | PP53 | + 181 | GCCAGGCATGCGTGCTTCGT |
| acetylglutamate kinase (*argB*) | GCGA | PP41 | + 11 | CGGCTACATGGGAAGCGGCA |
|  | AGG | PP54 | + 5 | GGGAAGCGGCATCGCGATCG |
| argininosuccinate lyase (*argH*) | GCGA | PP42 | + 76 | ATCTACCGAGGCGGTGAAAC |
|  | CGG | PP55 | + 18 | CCAGGACTGATTGGTCTTCT |
|  | AGG | PP56 | -3 | CTCATGGATTCACTCATTGC |
|  | GGG | PP57 | + 60 | CGGGCGACGAAGGCGTCGAC |
|  | TGG | PP58 | + 224 | TGCCGGCTTCAATCTCGCCC |
| 2-keto-3-deoxy-6-phosphogluconate aldolase (*eda*) | GTGA | PP43 | -20 | GGCACCAGTAGATGTCCAGGGGG |
|  | TGG | PP59 | + 19 | TTCAAGGGTGGTCATGACTG |
| phosphogluconate dehydratase (*edd*) | GTGA | PP44 | + 22 | GCTGCGTTCGACCAGCCGCT |
|  | CGG | PP60 | + 6 | TGGGTGACCTCAAGGATGCG |
|  | AGG | PP61 | - 12 | GGATGCATGTACTGGACTCC |
|  | GGG | PP62 | + 25 | GCTGCGTTCGACCAGCCGCT |
|  | GGG | PP63 | + 238 | GAAGTGCAAGTAAGGCTGGT |
| cell division protein (*ftsZ*) | GTGA | PP45 | + 83 | CTTCGATGCTGCTCTTGACC |
|  | CGG | PP64 | + 24 | ACTTTAATGACCGGACTTTG |
| chorismite mutase (*pheA*) | GCGA | PP46 | + 129 | ACCGGCTTCTCGCCTTCTTT |
|  | CGG | PP65 | + 13 | CAGCGCCTTGAGCTCCTGTT |
|  | AGG | PP66 | -50 | CCAGCGCTTCTACCGCTTCC |
|  | AGG | PP67 | + 53 | GCTCGAGAATTTTCTCGTCG |
|  | GGG | PP68 | + 215 | CCATCTCTTCGTTGCCCAGC |
| orotidine 5’-phosphate decarboxylase (*pyrF*) | GCGA | PP47 | + 28 | CTCACGGGTAGGGAAATCCA |
|  | AGG | PP69 | + 20 | GGGAAATCCAGGGCGACGATC |
|  | GGG | PP70 | -5 | CTGGCAGGCGGACATGGGCA |
|  | CGG | PP71 | + 4 | GATCAGGGGCGTCTGGCAGG |
|  | CGG | PP72 | + 238 | GACCATCCACACGCCCATCT |
| anthranilate synthase component 2 (*trpG*) | GTGA | PP48 | + 31 | AAGGTACTGAACAACGTTGT |
|  | TGG | PP73 | +1 | ATTGTCGATCATCAGTAACA |
|  | CGG | PP74 | -45 | ACTTCATGATTCGAACCTTC |
|  | CGG | PP75 | +70 | ATTGCGAATGACCTTGACCT |
|  | TGG | PP76 | +236 | GCCCGATTGACTGGTGGCCC |

**Supplementary Figures**


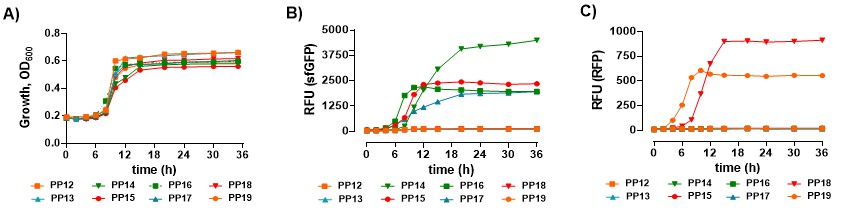


**Figure S1:** Kinetic data for recombinant strains harboring pRGPDuo3 and pRGPDuo4-derived vectors for OD600 **(A)**, sfGFP **(B)** and RFP **(C)**. sfGFP and RFP values are not normalized against OD_600_. All the strains were induced with IPTG (1 mM) and arabinose (0.2%). The results are representation of data from at least two independent cultivations.


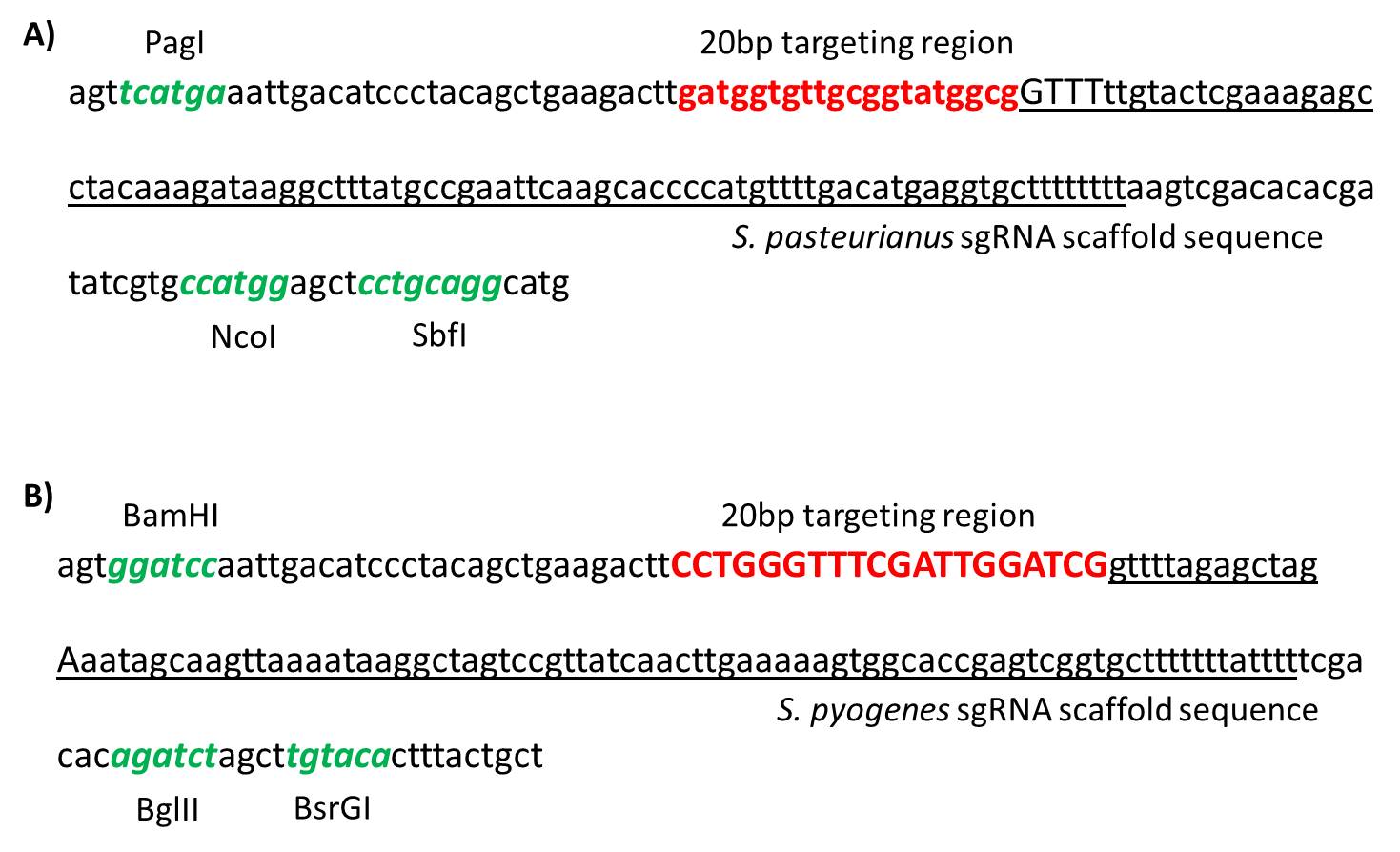


**Figure S2: Design of sgRNA coding sequences.** Example of sequence for designed synthetic fragment, to target specific gene using CRISPRi for pRGPdCas9bad **(A)** and pRGPspdCas9bad **(B)**. The sequence consist of 20 base pair gene targeting sequence followed by sgRNA scaffold sequence for dCas9 binding. The recognition sequences for restriction enzyme are specific for sgRNA cloning into CRISPRi vectors (pRGPdCas9bad and pRGPspdCas9bad). Multiplex gene targeting using these vectors require including of promoter sequence between targeting region and BamHI restriction site for each new sgRNA for concatenation.


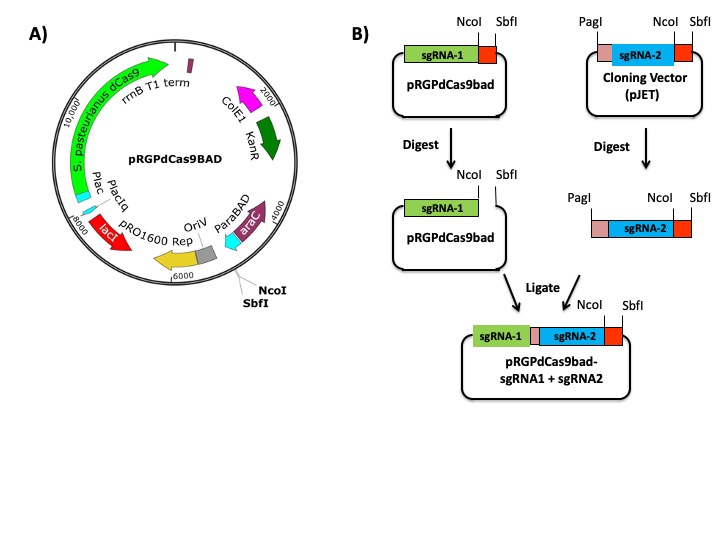


**Figure S3:** Vector map for pRGPdCas9bad **(A)**. Enzyme based sgRNA cloning strategy for CRISPRi vector pRGPdCas9bad (and pRGPdCas9) **(B)**. The sgRNA sequences were flanked with restriction site PagI to the 5’ end, and the NcoI and SbfI were introduced at the 3’ end. The sgRNA fragment can be first cloned into cloning vector such as pJET 1.2 blunt (optional step). For sgRNA cloning into CRISPRi vector, the insert was digested with PagI and SbfI and the CRISPRi vector was digested with NcoI and SbfI. The ligation of these fragments restore the original restriction sites (NcoI and SbfI) that can be further used for second round of sgRNA cloning. Therefore, the strategy allows concatenation of multiple sgRNAs in single vector for multiplex gene targeting. A similar strategy was used for cloning sgRNAs in *S. pyogenes* based CRISPRi vector where BglII/BsrGI digested pRGPspdCas9bad was ligated with BamHI/BsrGI digested sgRNA insert.


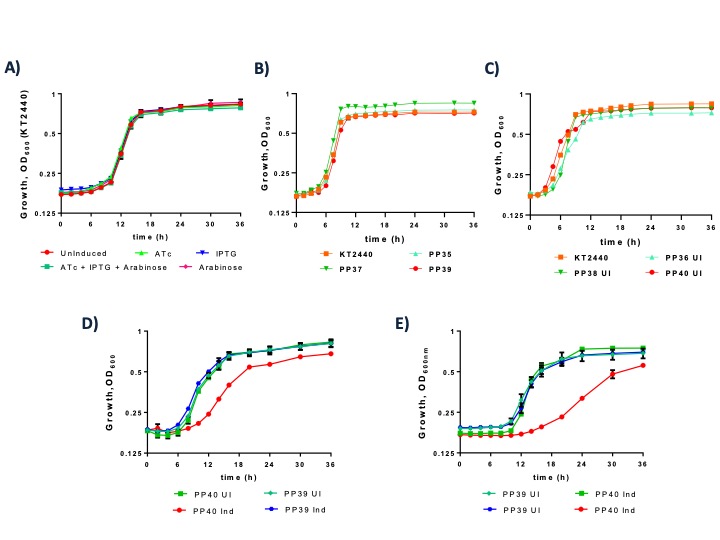


**Figure S4:** Growth analysis of *P. putida* KT2440 and its derivative strains in M9 minimal media. (A) The analysis of *P. putida* growth in the presence of different inducers when pre-cultures were not induced. (B) Growth comparison of *P. putida* strains carrying vectors pRGPsgRNA (PP35), pRGPdCas9 (PP37) and pRGPdCas9bad (PP39) with no sgRNA insert. (C) Growth comparison of *P. putida* strains expressing *aceE* targeting sgRNA when inducers were not added (UI means uniduced). (D) Growth comparison of *P. putida* strains expressing *aceE*-targeting sgRNA in the presence and absence of inducers when precultures were not induced. (E) Growth comparison of *P. putida* strains expressing *aceE*-targeting sgRNA in the presence and absence of inducers when precultures were also induced accordingly. Each graph represent the means and standard deviations of results from duplicate cultures. The results are representation of data from at least two independent cultivations.


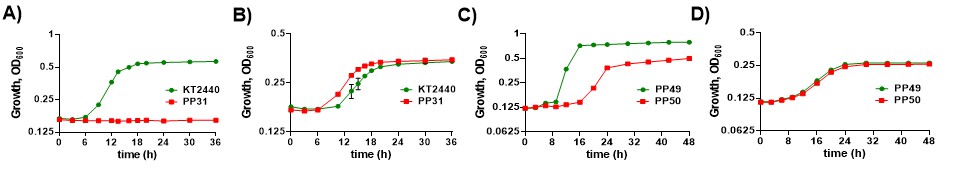


**Figure S5:** Growth analysis of *P. putida* KT2440 and its derivative strains in M9 minimal media with different substrates. Comparison of growth for *P. putida* KT2440 with deletion mutant strain PP31 (deletion of *aceEF* operon) in M9 media with glucose **(A)** and M9 media supplemented with *p*-coumaric acid (0.5%) **(B)**. Growth comparison of CRISPRi constructs PP49 (pRGPspdCas9bad) and PP50 (pRGPspdCas9bad-*aceE1*) in M9 glucose **(C)** and M9 *p*-coumaric acid **(D)** when pre-cultures were not induced.


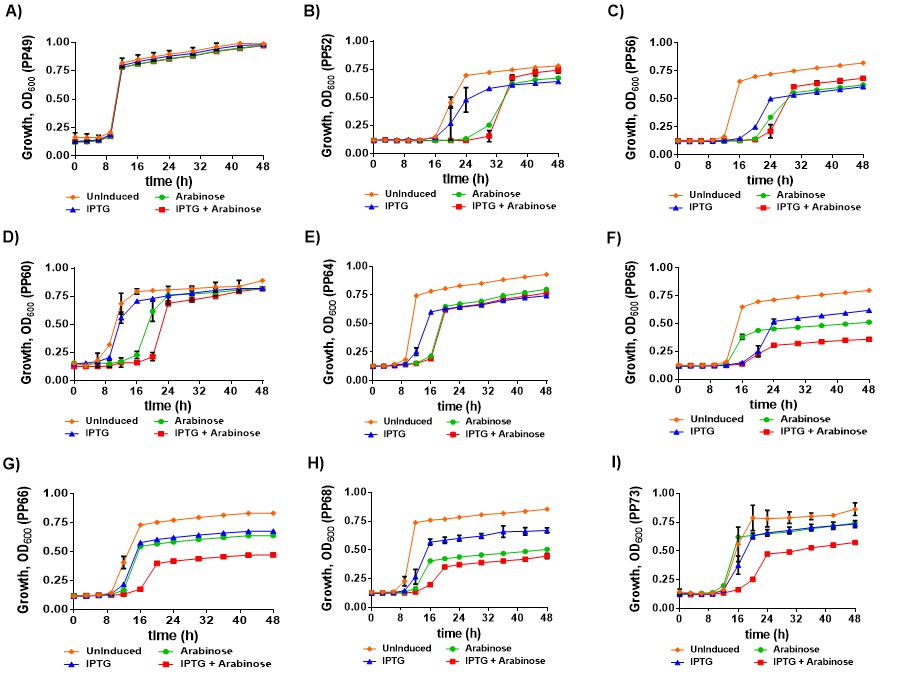


**Figure S6:** Growth analysis of *P. putida* KT2440 and its derivative strains in M9 minimal media. Leakiness of *S. pyogenes* spdCas9-based CRISPRi system (pRGPspdCas9bad) and its effect on growth when pre-cultures were not induced. Following recombinant strains were analyzed: PP49 **(A)**, PP52 **(B)**, PP56 **(C)**, PP60 **(D)**, PP64 **(E)**, PP65 **(F)**, PP66 **(G)**, PP68 **(H),** and PP73 **(I)**. For strains’ description refer **Table 1**. The annotation indicates the presence or absence of inducers: uninduced (no inducer added); induced (presence of both inducers: 1mM IPTG and 0.2 % w/v arabinose), + IPTG (the only IPTG was added), and + arabinose (the only arabinose was added). Each graph represents the mean values of biological triplicates from at least two individual cultivations, and error bars represent standard deviations.


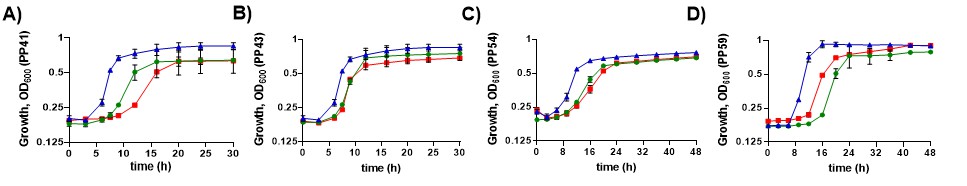


**Figure S7:** Comparison of gene targeting in *P. putida* KT2440 using *S. pasteurianus* and *S. pyogenes* based CRISPRi vectors pRGPdCas9bad **(A and B)** and pRGPspdCas9bad **(C and D)**, respectively. sgRNAs were designed to downregulate the expression of essential genes, namely, *argB* **(A and C)** and *eda* **(B and D)** by targeting the non-template strand. Growth phenotype was assessed for the strains PP41 (carrying pRGPdCas9bad-*argB*), PP43 (carrying pRGPdCas9bad-*eda*), PP54 (carrying pRGPspdCas9bad-*argB1*), PP59 (carrying pRGPspdCas9bad-*eda1*) and compared to the respective control strain PP39 (carrying pRGPdCas9bad) or PP49 (pRGPspdCas9bad). In each graph strain PP39 or PP49 is represented as a blue triangle. The green circle in each graph represents the respective strain when pre-cultures are not induced. The red square in each graph represents the respective strain when pre-cultures are induced. Each graph represents the mean values of biological triplicates from at least two individual cultivations, and error bars represent standard deviations.


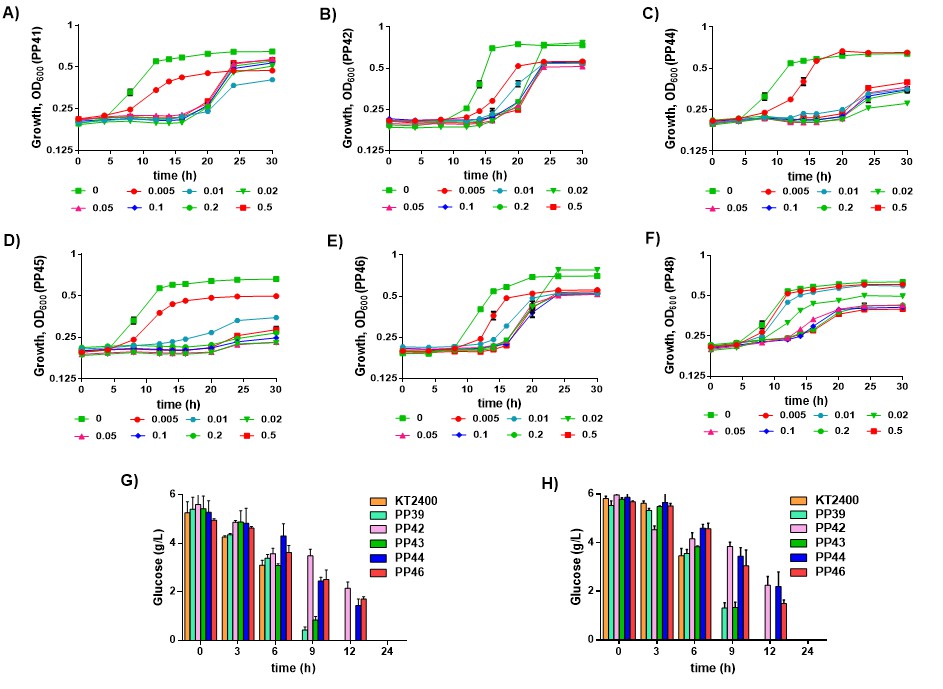


**Figure S8:** The CRISPRi-mediated tunable repression of essential genes in *P. putida* KT2440. The expression of dCas9 from *S. pasteurianus* is under *P_lac_* control (IPTG-inducible), and the strong pBAD promoter (arabinose-inducible) controls sgRNA expression. Growth of recombinant *P. putida* strains PP41 **(A)**, PP42 **(B)**, PP44 **(C)**, PP45 **(D)**, PP46 **(E)**, and PP48 **(F)** was investigated in the presence of different concentrations of arabinose (ranging from 0 to 0.5 % w/v). The dCas9 expression was induced by adding IPTG (1mM) in all strains. For the tunable experiment, pre-cultures were induced accordingly. Time course consumption of glucose was measured in *P. putida* strains KT2440, PP39, PP42, PP43, PP444, and PP46 under conditions when pre-cultures were not induced **(G)**, and when pre-cultures were induced with both inducers (1mM IPTG and 0.2 % arabinose) **(H)**. Each graph represents the means and standard deviations of results from duplicate cultures. The results are a representation of data from at least two independent cultivations.


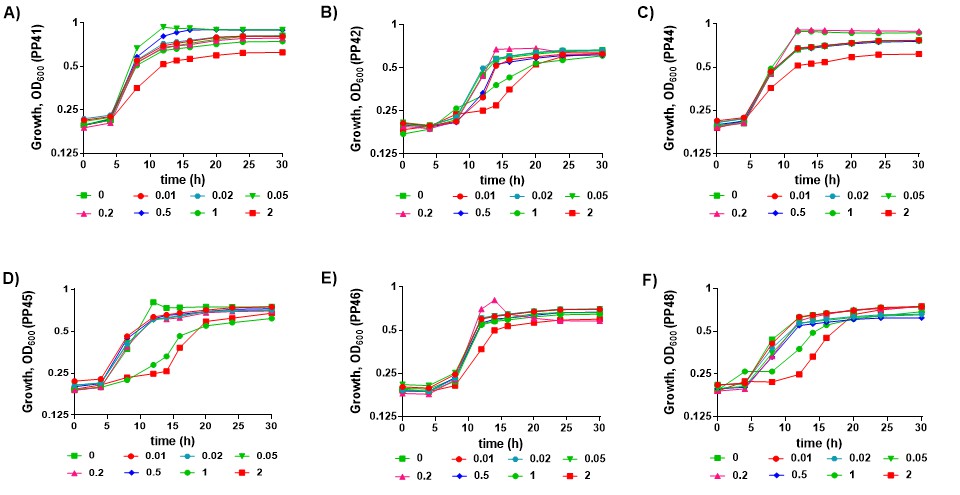


**Figure S9:** The CRISPRi-mediated tunable repression of essential genes in *P. putida* KT2440. The expression of dCas9 from *S. pasteurianus* is under *P_tac_* control (IPTG inducible) while the strong pBAD promoter (arabinose inducible) controls sgRNA expression. Growth of recombinant *P. putida* strains PP41 (carrying pRGPdCas9bad-*argB*), PP42 (carrying pRGPdCas9bad-*argH*), PP44 (carrying pRGPdCas9bad-*edd*), PP45 (carrying pRGPdCas9bad-*ftsZ*), PP46 (carrying pRGPdCas9bad-*pheA*), and PP48 (carrying pRGPdCas9bad-*pyrF*) was investigated in the presence of different concentrations of IPTG (ranging from 0 mM to 2 mM). The sgRNA expression targeting respective essential gene was induced by adding arabinose (0.2 % w/v). For tunable experiment precultures were induced accordingly. Each graph represent the means and standard deviations of results from duplicate cultures. The results are representation of data from at least two independent cultivations.
